# Elevated α-synuclein levels inhibit mitophagic flux

**DOI:** 10.1038/s41531-024-00696-0

**Published:** 2024-04-09

**Authors:** Inge Kinnart, Liselot Manders, Thibaut Heyninck, Dorien Imberechts, Roman Praschberger, Nils Schoovaerts, Catherine Verfaillie, Patrik Verstreken, Wim Vandenberghe

**Affiliations:** 1 Department of Neurosciences, Laboratory for Parkinson Research, KU Leuven, Leuven, Belgium; 2 Department of Neurosciences, Laboratory for Neuronal Communication, KU Leuven, Leuven, Belgium; 3 VIB-KU Leuven Center for Brain & Disease Research, Leuven, Belgium; 4 Stem Cell and Developmental Biology, KU Leuven, Leuven, Belgium; 5 Department of Neurology, University Hospitals Leuven, Leuven, Belgium

**Keywords:** Parkinson's disease, Cell biology

## Abstract

The pathogenic effect of *SNCA* gene multiplications indicates that elevation of wild-type α-synuclein levels is sufficient to cause Parkinson’s disease (PD). Mitochondria have been proposed to be a major target of α-synuclein-induced damage. PINK1/parkin/DJ-1-mediated mitophagy is a defense strategy that allows cells to selectively eliminate severely damaged mitochondria. Here, we quantified mitophagic flux and non-mitochondrial autophagic flux in three models of increased α-synuclein expression: 1/*Drosophila melanogaster* that transgenically express human wild-type and mutant α-synuclein in flight muscle; 2/human skin fibroblasts transfected with α-synuclein or β-synuclein; and 3/human induced pluripotent stem cell (iPSC)-derived neurons carrying an extra copy of wild-type *SNCA* under control of a doxycycline-inducible promoter, allowing titratable α-synuclein upregulation. In each model, elevated α-synuclein levels potently suppressed mitophagic flux, while non-mitochondrial autophagy was preserved. In human neurons, a twofold increase in wild-type α-synuclein was already sufficient to induce this effect. PINK1 and parkin activation and mitochondrial translocation of DJ-1 after mitochondrial depolarization were not affected by α-synuclein upregulation. Overexpression of the actin-severing protein cofilin or treatment with CK666, an inhibitor of the actin-related protein 2/3 (Arp2/3) complex, rescued mitophagy in neurons with increased α-synuclein, suggesting that excessive actin network stabilization mediated the mitophagy defect. In conclusion, elevated α-synuclein levels inhibit mitophagic flux. Disruption of actin dynamics may play a key role in this effect.

## Introduction

Compelling evidence indicates that α-synuclein plays a central role in the pathogenesis of Parkinson’s disease (PD). Aggregated α-synuclein is the main protein component of Lewy bodies^1^. Very rare missense mutations in *SNCA*, the gene encoding α-synuclein, and more common multiplications (duplications or triplications) of the wild-type *SNCA* locus cause autosomal dominant PD^2^. The pathogenic effect of *SNCA* multiplications implies that elevation of wild-type α-synuclein levels is sufficient to cause PD. In addition, polymorphisms in regulatory elements of *SNCA* are risk factors for sporadic PD^2^. Interestingly, α-synuclein protein levels increase in human nigral dopaminergic neurons during normal aging, which could potentially explain why aging is a strong risk factor for PD^3^.

α-Synuclein is a 140 amino acid neuronal protein enriched in presynaptic terminals^4^. In pathological conditions, α-synuclein can misfold and aggregate to form oligomers, protofibrils, and insoluble fibrils^5^. Misfolded α-synuclein can spread between neurons and promote further α-synuclein misfolding in a prion-like fashion^6^. Although pathological α-synuclein may cause neurotoxicity through disruption of multiple cellular pathways, accumulating evidence suggests that induction of mitochondrial damage is one of the main mechanisms^7^. Pathological α-synuclein species have been shown to have multiple damaging effects on mitochondria, such as impairment of electron transport chain complexes, interference with mitochondrial protein import, and disruption of mitochondria-associated membranes^8–12^. In neurons, seeding events for α-synuclein aggregation occur preferentially on mitochondrial membranes, where these aggregates impair complex I activity, increase mitochondrial reactive oxygen species (ROS) generation, and induce mitochondrial permeability transition and cell death^13^.

One of the most drastic defense lines cells have developed to protect themselves against life-threatening accumulation of damaged mitochondria is mitophagy^14^. Mitophagy is a mitochondrial quality control pathway in which damaged mitochondria are selectively engulfed by autophagosomes and then destroyed inside lysosomes. Of direct relevance to PD, one particular mitophagic pathway critically depends on PINK1, parkin, and DJ-1, three proteins whose functions are disrupted by autosomal recessive PD mutations^15–17^. In this pathway, the ubiquitin and parkin kinase PINK1 selectively accumulates on the outer mitochondrial membrane (OMM) of damaged mitochondria and activates parkin to ubiquitinate multiple OMM proteins^14–16^. In neurons, the autophagy receptor optineurin is recruited to mitochondrial ubiquitin chains in a DJ-1-dependent manner and connects ubiquitin to LC3 present on autophagosomal membranes, thus promoting encapsulation of defective mitochondria by autophagosomes^17^. Intriguingly, recent studies revealed that Lewy bodies in PD brains do not only contain insoluble α-synuclein and other proteins but also large amounts of lipids derived from damaged mitochondria and autophagosomal and lysosomal membranes, suggesting that pathological α-synuclein may interfere with mitophagy^18^.

Here, we investigated whether elevated levels of wild-type α-synuclein have any effect on mitophagy. Our findings in *Drosophila* flight muscle in vivo and in cultured human fibroblasts and neurons indicate that increased α-synuclein abundance blocks mitophagy and thus obstructs the ability of cells to mount this ultimate defensive response against accumulation of defective mitochondria.

## Results

### α-Synuclein inhibits mitophagic flux in *Drosophila*

**Fig. 1:**
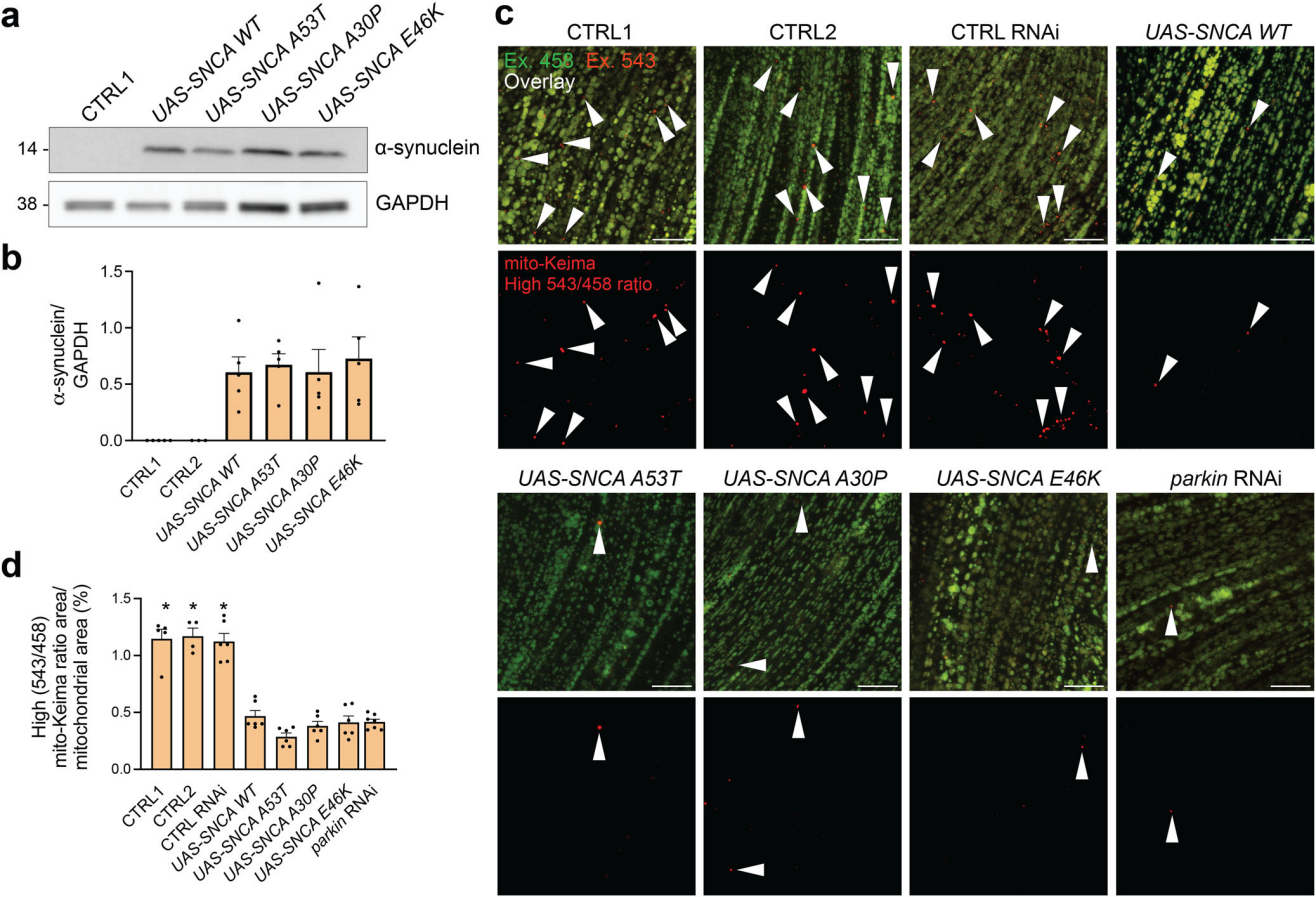
Mitophagic flux is impaired in *Drosophila* expressing human wild-type or mutant α-synuclein. **a** Western blot for α-synuclein on extracts from thoraces of 4-week-old flies expressing wild-type or mutant human α-synuclein (*w1118;;UAS-mito-Keima,mef-2-GAL4/UAS-SNCA X*) and control flies (CTRL1: *w1118;;UAS-mito-Keima,mef-2-GAL4/+*). **b** Quantification of the α-synuclein/GAPDH ratio in (**a**) (*n* = 5 for all conditions, except for CTRL2 where *n* = 3; *P* = 0.94, one-way ANOVA with post hoc Tukey’s test). CTRL2 flies are *w1118;;UAS-mito-Keima,mef-2-GAL4/UAS-smGdP*. **c** Confocal images showing overlay of live mito-Keima emission at 458 nm (green) and 543 (red) excitation in indirect flight muscle of 4-week-old CTRL1, CTRL2, CTRL RNAi (*w1118; UAS-Ctrl RNAi/+; UAS-mito-Keima, mef-2-GAL4*), wild-type and mutant α-synuclein (*w1118;;UAS-mito-Keima,mef-2-GAL4/UAS-SNCA X*) and parkin RNAi (*w1118; UAS-parkin RNAi/+; UAS-mito-Keima, mef-2-GAL4/+*) flies. High 543/458 ratio signal puncta correspond to mito-Keima present in lysosomes, indicated by *arrowheads*. Scale bar, 10 µm. **d** High mito-Keima (543/458) ratio area/total mitochondrial area was quantified as an index of mitophagic flux (*n* = 5–6 flies per condition, from at least 3 different crosses). In each fly, 10 random 2500 μm^2^ fields were analyzed. One-way ANOVA with post hoc Tukey’s test. *P < 0.0001 compared to all flies expressing α-synuclein and parkin RNAi flies. Error bars represent SEM.

We previously engineered a fly model that transgenically expresses the sensitive mitophagy reporter mito-Keima, allowing detection of mitophagic flux in vivo in the absence of exogenous mitochondrial toxins^19^. Mito-Keima is a mitochondrially targeted form of Keima, a fluorescent protein that is resistant to lysosomal proteases and exhibits pH-dependent excitation^20^. The peak of the excitation spectrum of mito-Keima shifts when mitochondria are delivered to the acidic lysosomal lumen, allowing live dual-excitation ratiometric quantification of mitophagic flux^20^. Using these mito-Keima flies we previously demonstrated an age-dependent increase in mitophagy in flight muscle, which was abrogated by parkin or PINK1 deficiency^19^. To determine the effect of α-synuclein on PINK1/parkin-dependent mitophagy in vivo, we transgenically expressed wild-type and 3 different PD-associated mutant (A30P, A53T, and E46K) forms of human α-synuclein in flight muscle of mito-Keima flies. Protein expression levels were similar for wild-type and mutant forms of α-synuclein (Fig. 1a, b). We measured mitophagy in flight muscle at the age of 4 weeks, a time point at which PINK1/parkin-dependent mitophagy is robustly detectable^19^. Interestingly, both wild-type and mutant α-synuclein potently suppressed mitophagy, to the same extent as parkin deficiency (Fig. 1c, d). There was no significant difference in the magnitude of mitophagy inhibition between wild-type and mutant α-synuclein.

**Fig. 2:**
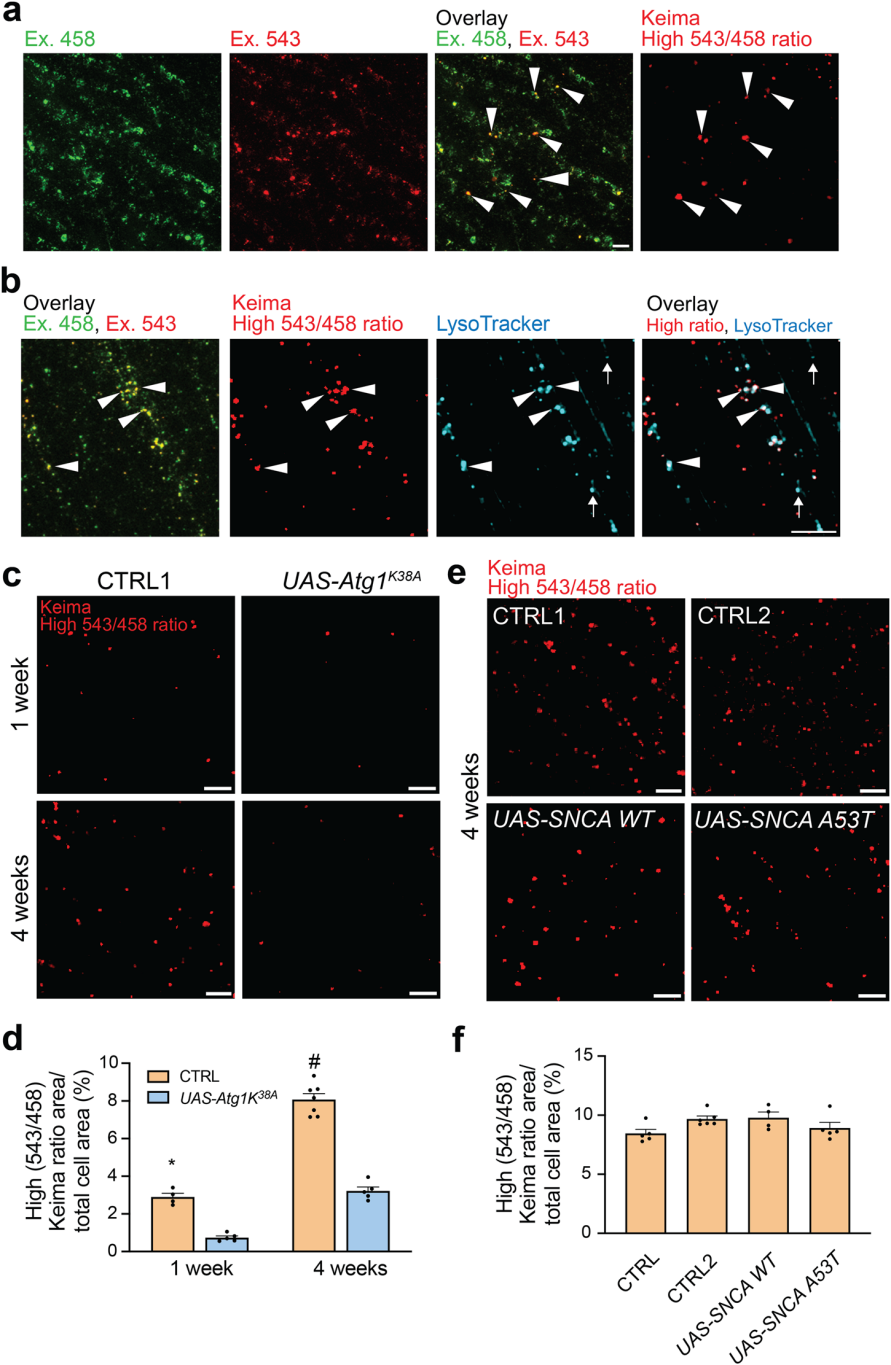
Non-mitochondrial autophagic flux is preserved in *Drosophila* expressing human wild-type or mutant α-synuclein. **a** Confocal images of Keima emission in indirect flight muscle of 4-week-old control flies (CTRL1: *w1118;;UAS-Keima,mef-2-GAL4/+*) at 458 nm (green) and 543 nm (red) excitation. *Arrowheads* indicate ‘acidic’ puncta with high 543/458 ratio values. **b** Confocal images of Keima-expressing indirect flight muscle of 4-week-old CTRL1 labeled with LysoTracker (100 nM), showing colocalization of ‘acidic’ Keima puncta with lysosomes (*arrowheads*). *Arrows* indicate examples of lysosomes without Keima signal. Additional images of colocalization of high 543/458 ratio Keima puncta with LysoTracker are shown in Supplementary Fig. 1. **c** High Keima (543/458) ratio signal of 1- and 4-week-old CTRL1 or Atg1^K38A^-overexpressing indirect flight muscle tissue. **d** Quantification of (**c**). High Keima (543/458) ratio area/ total cell area was quantified as an index of non-mitochondrial autophagic flux (*n* = 5−6 flies per condition, from at least 3 different crosses). One-way ANOVA with post hoc Tukey’s test. *P < 0.0001 compared with 1-week-old Atg1^K38A^ flies and 4-week-old CTRL1 flies. #*P* < 0.0001 compared with 4-week-old Atg1^K38A^ flies. **e** High Keima (543/458) ratio signal in indirect flight muscle of 4-week-old CTRL1 and CTRL2 (*w1118;;UAS-Keima,mef-2-GAL4/UAS-smGdP*) flies and flies expressing wild-type (*w1118;;UAS-Keima,mef-2-GAL4/UAS-SNCA WT*) or mutant A53T α-synuclein (*w1118;;UAS-Keima,mef-2-GAL4/UAS-SNCA A53T)*. **f** Quantification of (**e**) (*n* = 5 flies per condition, from at least 3 different crosses; *P* = 0.16, one-way ANOVA with post hoc Tukey’s test). Error bars represent SEM. Scale bars, 10 µm.

To assess whether α-synuclein also induced a more general impairment of macro-autophagy, we generated flies that transgenically express Keima. Keima is the same reporter as mito-Keima except that it lacks the mitochondrial targeting sequence and is therefore targeted to non-mitochondrial compartments, primarily the cytosol^20^. Live imaging in flight muscle of Keima flies showed that a subset of Keima structures had high 543 nm/458 nm ratio values, indicative of an acidic environment (Fig. 2a). The vast majority (90.8 ± 1.2%; *n* = 3) of these high 543/458 ratio Keima puncta colocalized with the lysosomal dye LysoTracker (Fig. 2b; Supplementary Fig. 1). The abundance of these puncta significantly increased between the age of 1 and 4 weeks (Fig. 2c, d). To assess whether the biogenesis of the high 543/458 ratio Keima puncta required autophagy, a kinase-dead version of Atg1 (Atg1^K38A^), the homolog of mammalian ULK1, was overexpressed. Atg1/ULK1 is needed in the early steps of autophagosome formation^21,22^, and kinase-dead Atg1 exerts dominant-negative effects^23^. Overexpression of Atg1^K38A^ indeed diminished formation of high 543/458 ratio Keima puncta (Fig. 2c, d). Surprisingly, expression of wild-type or A53T mutant α-synuclein had no effect on the abundance of high 543/458 ratio Keima puncta in 4-week-old flight muscle (Fig. 2e, f), indicating that these flies had no general macro-autophagy defect.

### α-Synuclein inhibits mitophagic flux in human skin fibroblasts

**Fig. 3:**
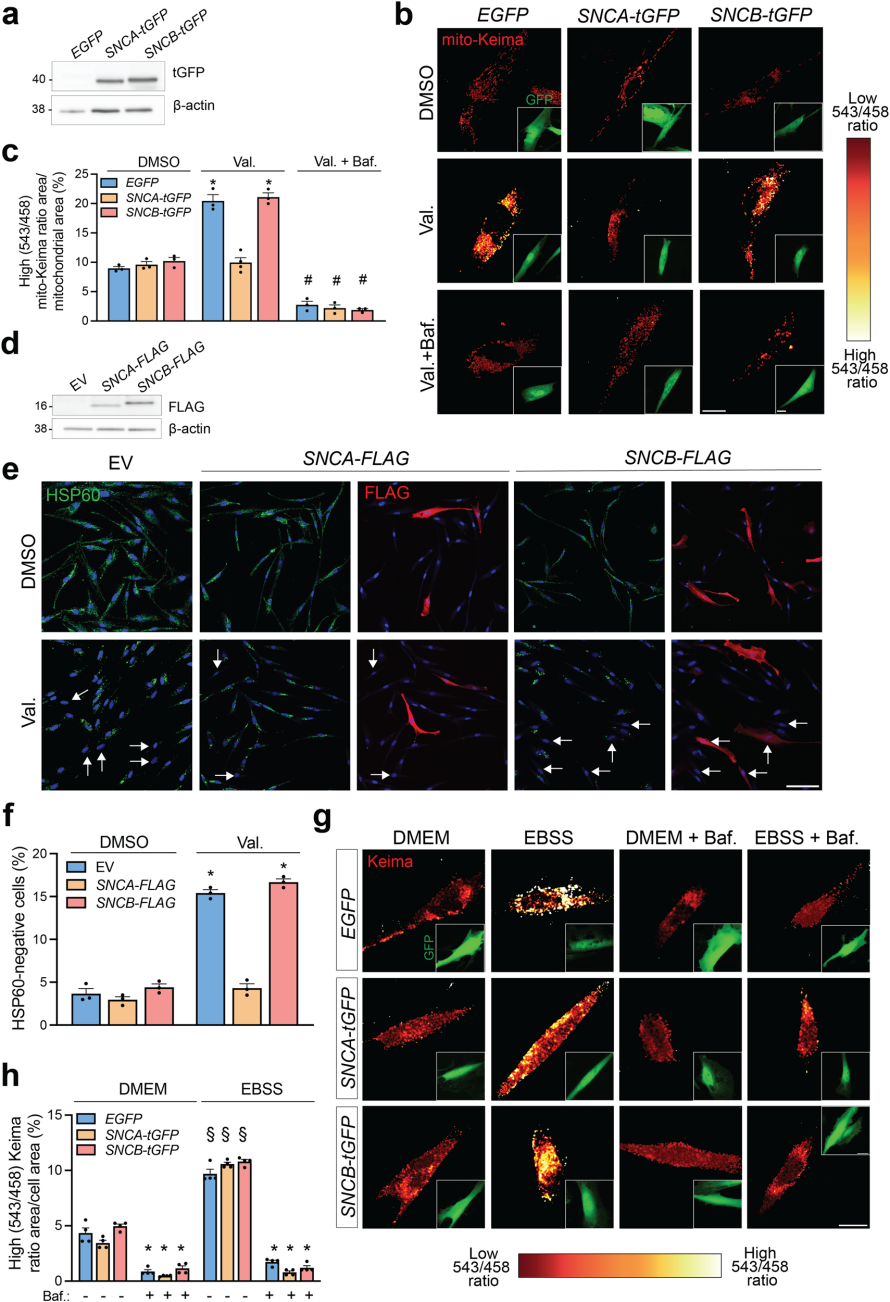
Overexpression of α-synuclein impairs mitophagy in human skin fibroblasts. **a** Western blot for TurboGFP (tGFP) and β-actin on extracts from human control fibroblasts transfected with EGFP (which is not recognized by anti-tGFP antibody), tGFP-tagged α-synuclein (*SNCA-tGFP*) or tGFP-tagged β-synuclein (*SNCB-tGFP*). **b** Fibroblasts were transfected with mito-Keima in combination with *EGFP, SNCA-tGFP* or *SNCB-tGFP* and treated with DMSO, valinomycin (Val., 1 μM) or a combination of Val. and the lysosomal vacuolar-type H^+^-ATPase inhibitor bafilomycin A1 (Baf., 100 nM) for 48 h, followed by live mito-Keima imaging. Scale bar, 10 µm. **c** High mito-Keima (543/458) ratio area/total mitochondrial area was quantified as an index of mitophagy (*n* = 3−6 experiments, with at least 15 cells analyzed per experiment for each condition). One-way ANOVA with post hoc Tukey’s test. *P < 0.0001 compared with DMSO- and Val.+Baf.-treated cells transfected with the same cDNA and compared with Val.-treated cells transfected with *SNCA-tGFP*. #*P* < 0.0001 compared with DMSO- and Val.-treated cells transfected with the same cDNA. **d** Western blot of control fibroblasts transfected with empty vector (EV), FLAG-tagged α-synuclein (*SNCA-FLAG*) or FLAG-tagged β-synuclein (*SNCB-FLAG*). **e** Control fibroblasts transfected with EV, *SNCA-FLAG*, or *SNCB-FLAG* were treated with DMSO or Val. for 48 h and immunostained for mitochondrial matrix protein HSP60. Nuclei were stained with TOTO-3 (blue). *Arrows* indicate examples of cells without detectable HSP60 staining. Scale bar, 20 µm. Additional images of this experiment are shown in Supplementary Fig. 2. **f** Quantification of % cells lacking detectable HSP60 as in (**e**) (*n* = 3 experiments, with at least 150 cells analyzed per experiment for each condition). The data for the EV condition represent all cells in that condition, whereas analysis of the *SNCA-FLAG* and *SNCB-FLAG* conditions was limited to FLAG-positive cells. One-way ANOVA with post hoc Tukey’s test. *P < 0.0001 compared with all DMSO-treated conditions and the Val.-treated *SNCA-FLAG* condition. **g** Fibroblasts were transfected with Keima in combination with *EGFP*, *SNCA-tGFP*, or *SNCB-tGFP* and incubated for 4 h in normal growth medium (DMEM) or growth medium lacking amino acids (EBSS) with or without bafilomycin A1 (Baf., 100 nM), followed by live Keima imaging. High (543/458) ratio signal corresponds to Keima present in lysosomes. Scale bar, 10 µm. **h** High Keima (543/458) ratio area**/**total cell area was quantified as an index of non-mitochondrial autophagy (*n* = 3 experiments, with at least 15 cells analyzed per experiment for each condition). One-way ANOVA with post hoc Tukey’s test. *P < 0.0001 compared with all conditions without Baf. §*P* < 0.0001 compared with all DMEM conditions. Error bars represent SEM.

Next, we assessed the effect of α-synuclein on mitophagy in cultured human skin fibroblasts from a healthy human control. We were unable to detect endogenous α-synuclein in these cells on western blot, in line with previous work^24,25^. We therefore transiently transfected the fibroblasts with TurboGFP-tagged α-synuclein and, as controls, with EGFP and TurboGFP-tagged β-synuclein, which is closely related to α-synuclein but has no established pathological or genetic link with PD^4^. We previously showed that exposure of fibroblasts to the mitochondrial uncoupler valinomycin induces mitophagy that is abrogated by deficiency of PINK1, parkin, or DJ-1^17,26^. Remarkably, overexpression of α-synuclein inhibited valinomycin-induced mitophagy, as measured by live mito-Keima imaging, while β-synuclein, despite similar overexpression levels, had no effect (Fig. 3a–c). We confirmed this finding by measuring valinomycin-induced clearance of the mitochondrial matrix protein HSP60 by immunostaining of fibroblasts transfected with empty vector and FLAG-tagged α- or β-synuclein (Fig. 3d–f; Supplementary Fig. 2).

We used live Keima imaging to assess whether overexpression of α-synuclein also interfered with non-mitochondrial autophagy. Exposure of fibroblasts to amino acid starvation, a classical trigger for non-selective autophagy, induced the emergence of high 543 nm/458 nm ratio Keima puncta that nearly all (91.7 ± 0.4%; *n* = 3) colocalized with LysoTracker and were suppressed by treatment with the lysosomal vacuolar-type H^+^-ATPase inhibitor bafilomycin A_1_ (Fig. 3g, h; Supplementary Fig. 3). Starvation-induced autophagy was unaffected by overexpression of α- or β-synuclein (Fig. 3g, h).

### Increased levels of α-synuclein inhibit mitophagic flux in human iPSC-derived neurons

**Fig. 4:**
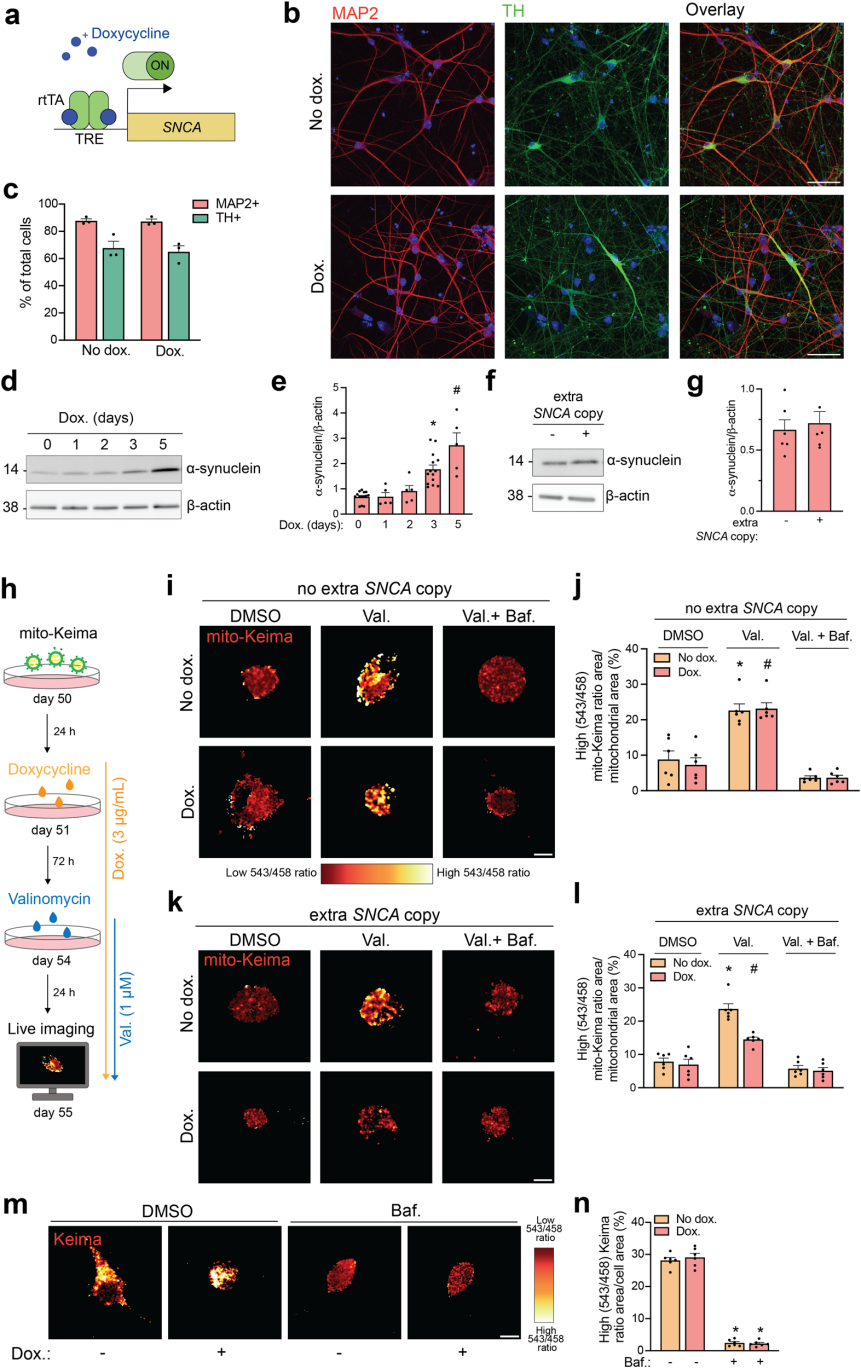
Elevated α-synuclein levels impair mitophagic flux in human iPSC-derived neurons without inducing a general autophagy defect. **a** Schematic showing the doxycycline-inducible extra *SNCA* copy inserted in a control iPSC line, resulting in α-synuclein upregulation when doxycycline is added to the medium. **b** iPSC-derived neuronal cultures carrying the doxycycline-inducible extra *SNCA* copy were treated with vehicle or doxycycline (dox., 3 μg/mL) for 3 days on day 50 after neuronal induction and immunostained for neuronal marker MAP2 and dopaminergic marker tyrosine hydroxylase (TH). Nuclei were stained with TOTO-3 (blue). Scale bar, 50 µm. **c** Percentage of MAP2- and TH-positive cells (*n* = 3). **d** Western blot for α-synuclein after treatment with dox. (3 μg/mL) for the indicated time period. **e** Quantification of (**d**) (*n* = 10). One-way ANOVA with post hoc Tukey’s test. *P = 0.0026 and ^#^*P* < 0.0001 compared to vehicle-treated cells. **f** Western blot for α-synuclein in iPSC-derived neurons with and without the inducible extra *SNCA* copy (both conditions without dox.). **g** Quantification of (**f**) (*n* = 3, *P* = 0.68, Student’s *t-*test). **h** Diagram of experimental design in (**i**–**l**). **i**–**l** Mito**-**Ke**i**ma live imaging was performed in iPSC-derived neurons without (**i**, **j**) or with (**k**, **l**) the extra dox.-inducible *SNCA* copy. Valinomycin (Val., 1 μM), bafilomycin A1 (Baf., 100 nM) and dox. (3 μg/mL, 3 days) were added as indicated. **j** Quantification of (**i**). High (543/458) ratio area/total mitochondrial area was quantified as an index of mitophagy (*n* = 6 experiments, each from a different differentiation, with at least 15 cells analyzed per condition per experiment). One-way ANOVA with post hoc Tukey’s test. *P < 0.0001 compared with DMSO- and Val.+Baf.-treated cells without dox. ^#^*P* < 0.0001 compared with DMSO- and Val.+Baf.-treated cells with dox. **l** Quantification of (**k**) (*n* = 6 experiments, each from a different differentiation, with at least 15 cells analyzed per condition per experiment). One-way ANOVA with post hoc Tukey’s test. *P < 0.0005 compared with DMSO- and Val.+Baf.-treated cells without dox. ^#^*P* < 0.005 compared with Val.-treated cells without dox. **m** iPSC-derived neuronal cultures were transduced with Keima lentivirus and treated with dox. or vehicle for 3 days, followed by live ratiometric imaging. **n** High (543/458) ratio area/total cell area was quantified as an index of non-mitochondrial autophagy (*n* = 6 experiments from 6 different differentiations, with at least 15 cells analyzed per condition per experiment). One-way ANOVA with post hoc Tukey’s test. *P < 0.0001 compared to the conditions without Baf. Error bars represent SEM. Scale bar in (**i**, **k**, **m**) 10 µm.

Next, we investigated the impact of elevated wild-type α-synuclein levels on mitophagy in iPSC-derived human neurons. We inserted an extra copy of the wild-type human *SNCA* gene into the AAVS1 safe harbor locus in a healthy human control iPSC line. This extra *SNCA* copy is under control of a doxycycline-inducible promoter (Fig. 4a). Using a previously established dopaminergic neuronal differentiation protocol^17^, we generated iPSC-derived cultures in which ~80% of all cells on day 50 after neuronal induction were neurons (based on MAP2 immunostaining) and ~60% of all cells were dopaminergic neurons (based on tyrosine hydroxylase [TH] immunostaining) (Fig. 4b, c). Addition of doxycycline (3 μg/mL) to the differentiated neurons for various time periods allowed titratable upregulation of wild-type α-synuclein (Fig. 4d, e). Doxycycline exposure for 72 h led to an approximately twofold increase of α-synuclein protein compared with endogenous levels (Fig. 4d, e). Without doxycycline exposure, α-synuclein levels were similar between neurons with versus without the doxycycline-controlled extra *SNCA* copy, indicating that α-synuclein expression from the extra *SNCA* cassette was not ‘leaky’ (Fig. 4f, g). This human neuronal model of increased α-synuclein expression had several advantages. First, the control neurons (i.e., neurons harboring the doxycycline-controlled extra *SNCA* copy without doxycycline exposure) were isogenic with the doxycycline-treated neurons. Second, batch-to-batch variability in neuronal differentiation from iPSCs did not confound our comparison of doxycycline-treated and -untreated neurons, because neurons in both conditions differentiated side by side from the same iPSC clone and only differed from each other in the doxycycline treatment on day 50 after neuronal induction. Third, this model allowed to assess the effect of increased α-synuclein more selectively than in iPSC-derived neurons from *SNCA* multiplication patients, because so-called *SNCA* multiplications are typically multiplications of large genomic regions that contain many other genes in addition to *SNCA*^27–29^. Immunostaining for cleaved caspase-3 and TH after upregulation of α-synuclein with doxycycline for 72 h showed that the percentage of apoptotic cells among dopaminergic neurons was very low (0.44 ± 0.4%; *n* = 3) and similar to the percentage without doxycycline treatment (0.48 ± 0.5%; *n* = 3).

We used lentiviral transduction to express mito-Keima in the iPSC-derived neurons for live mitophagy imaging (Fig. 4h). We previously showed that valinomycin treatment of iPSC-derived neurons induced mitophagy that was suppressed by the lysosomal vacuolar-type H^+^-ATPase inhibitor bafilomycin A1, by 3-methyladenine, an inhibitor of macro-autophagy induction, and by parkin or DJ-1 deficiency^17^. Importantly, ~twofold upregulation of wild-type α-synuclein (induced by 72 h doxycycline treatment) was sufficient to block valinomycin-induced mitophagy (Fig. 4k, l). Doxycycline treatment of neurons without doxycycline-controlled *SNCA* copy had no effect on mitophagy (Fig. 4i, j), indicating that doxycycline itself did not inhibit mitophagy. Flux of basal non-mitochondrial autophagy, as measured with Keima imaging, was not inhibited by upregulation of α-synuclein (Fig. 4m, n). Taken together, this suggested that elevated wild-type α-synuclein impaired mitophagy in iPSC-derived neurons without causing a general autophagy defect.

### Upregulation of α-synuclein in human neurons does not interfere with PINK1, parkin or DJ-1 function

**Fig. 5:**
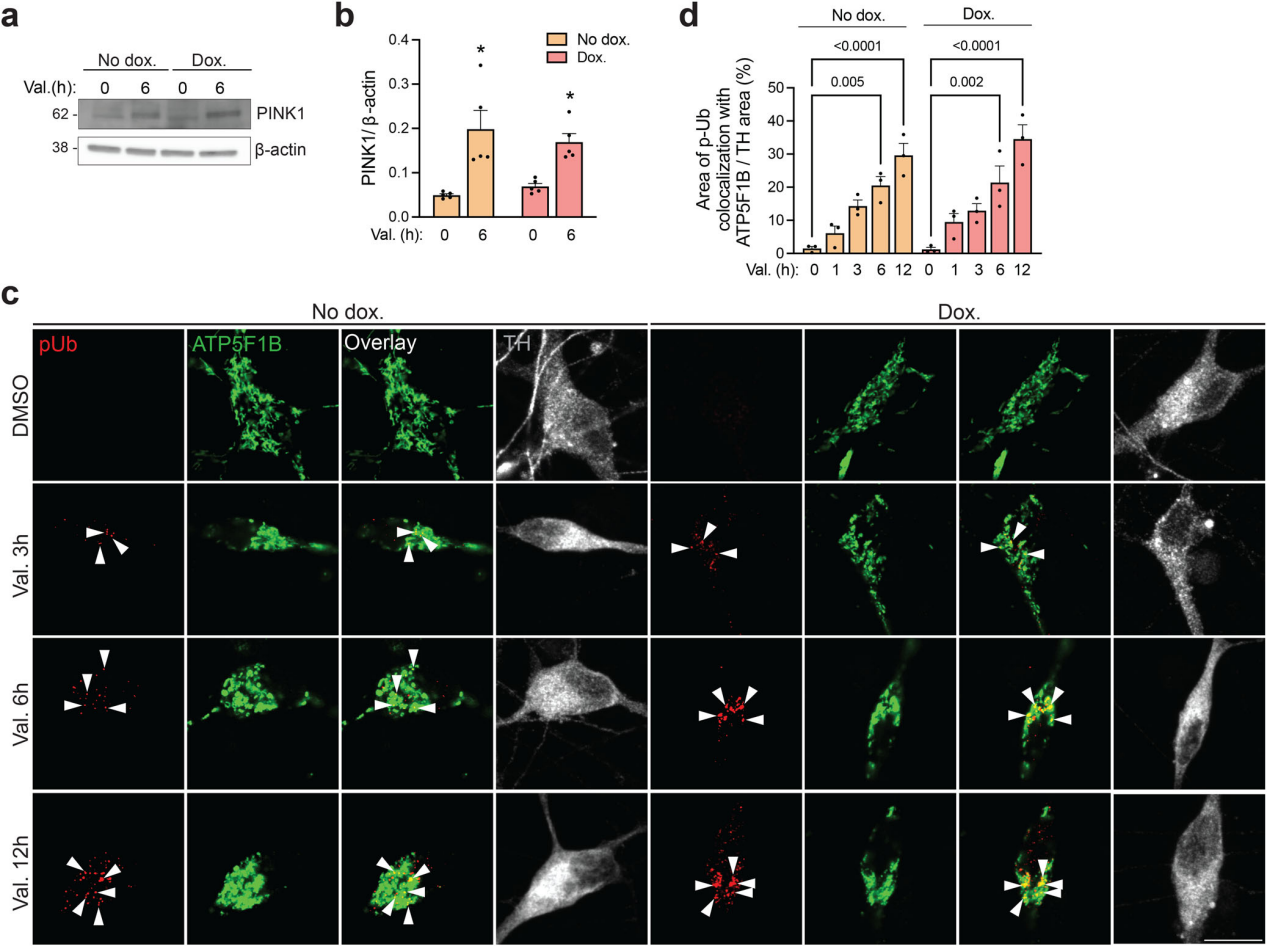
Upregulation of α-synuclein in human neurons does not interfere with PINK1 function. **a** iPSC-derived neurons carrying the doxycycline (dox.)-inducible extra *SNCA* copy with or without dox. treatment (3 μg/mL, 3 days) were exposed to valinomycin (Val., 1 μM) or DMSO for 6 h, followed by western blot for endogenous PINK1 and β-actin. **b** Quantification of (**a**) (*n* = 5, each from a different differentiation). One-way ANOVA with post hoc Tukey’s test. *P < 0.05 compared to cells without val. treatment and with the same respective Dox. or No dox. treatment. **c** iPSC-derived neurons carrying the dox.-inducible extra *SNCA* copy with or without dox. treatment (3 μg/mL, 3 days) were exposed to val. (1 μM) or DMSO for the indicated time periods, followed by immunostaining for endogenous phospho-ubiquitin (p-Ub), mitochondrial marker ATP5F1B, and tyrosine hydroxylase (TH). *Arrowheads* indicate examples of colocalization of p-Ub puncta with mitochondria. Scale bar, 10 µm. **d** Area of colocalization of p-Ub with ATP5F1B relative to the TH-positive area was quantified per TH-positive cell (*n* = 3 experiments, each from a different differentiation; at least 20 neurons analyzed per experiment for each condition). One-way ANOVA with post hoc Tukey’s test. Significant differences are indicated with their *P*-values. Error bars represent SEM.

Next, we asked whether the mitophagy defect in neurons with increased α-synuclein levels was due to disrupted function of PINK1, parkin, or DJ-1. However, accumulation of endogenous PINK1 after valinomycin treatment did not differ between dopaminergic neurons with and without α-synuclein upregulation (Fig. 5a, b). Also, formation of phospho-ubiquitin, the reaction product of PINK1, on depolarized mitochondria (Fig. 5c, d) was unaffected by α-synuclein upregulation. A small minority of phospho-ubiquitin puncta after valinomycin treatment appeared to be localized in the nucleus (both with and without doxycycline treatment) (Fig. 5c), consistent with a previous report of PINK1-mediated phospho-ubiquitin accumulation in the nucleus after mitochondrial depolarization^30^.

**Fig. 6:**
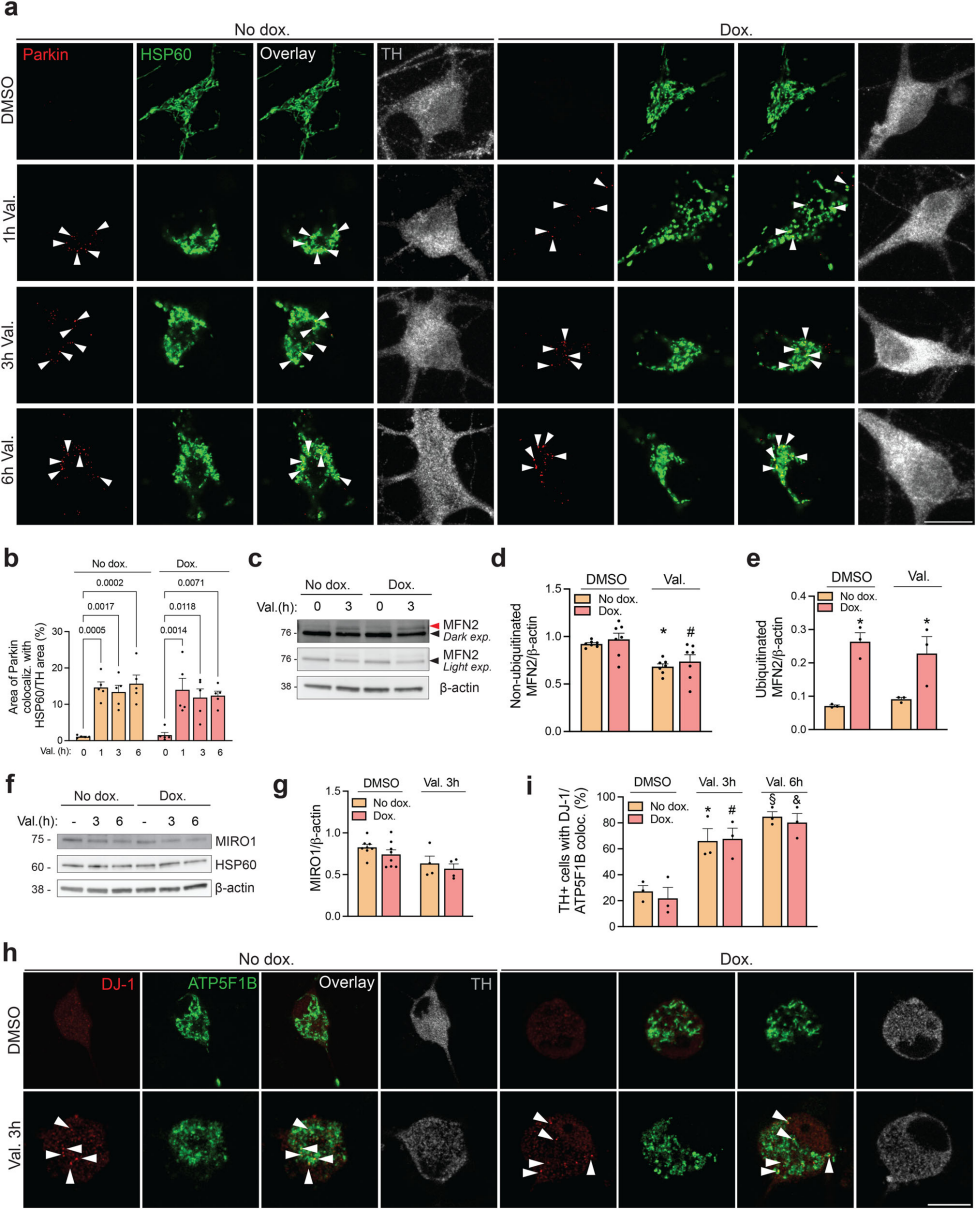
Upregulation of α-synuclein in human neurons does not interfere with mitochondrial translocation and activation of parkin or mitochondrial translocation of DJ-1. **a** iPSC-derived neurons carrying the doxycycline (dox.)-inducible extra *SNCA* copy with or without dox. treatment (3 μg/mL, 3 days) were exposed to valinomycin (Val., 1 μM) or DMSO for the indicated time periods, followed by immunostaining for endogenous parkin, mitochondrial marker HSP60 and tyrosine hydroxylase (TH). *Arrowheads* indicate examples of colocalization of parkin puncta with mitochondria. **b** The area of colocalization of parkin with HSP60 relative to the TH-positive area was quantified per TH-positive cell (*n* = 5 experiments, each from a different differentiation; at least 20 neurons analyzed per experiment for each condition). One-way ANOVA with post hoc Tukey’s test. Significant differences are indicated with their *P*-values. **c** Neurons carrying the dox.-inducible *SNCA* copy were treated with dox. or vehicle for 3 days, followed by Val. treatment for 3 h and western blot for endogenous mitofusin 2 (MFN2). The same MFN2 blot is shown after light or dark exposure (exp.). *Black arrowhead* indicates non-ubiquitinated MFN2 and *red arrowhead* indicates ubiquitinated MFN2. **d** Quantification of non-ubiquitinated MFN2 relative to β-actin (*n* = 7, each from a different differentiation). One-way ANOVA with post hoc Tukey’s test. *P = 0.01 compared to DMSO-treated cells without dox. ^#^*P* = 0.02 compared to DMSO-treated cells with dox. **e** Quantification of ubiquitinated MFN2 relative to β-actin (*n* = 3, each from a different differentiation). One-way ANOVA with post hoc Tukey’s test. *P < 0.05 compared to No dox. and the same respective DMSO or Val. treatment. **f** Neurons carrying the dox.-inducible *SNCA* copy were treated with dox. or vehicle for 3 days, followed by Val. treatment for the indicated time and western blot for endogenous MIRO1. **g** Quantification of MIRO1 relative to β-actin (*n* = 7, each from a different differentiation). **h** iPSC-derived neurons carrying the dox.-inducible *SNCA* copy with or without dox. treatment were exposed to Val. or DMSO for 3 h, followed by immunostaining for endogenous DJ-1, ATP5F1B, and TH. *Arrowheads* indicate colocalization of DJ-1 puncta with mitochondria. **i** Quantif**i**cation of % TH-positive cells with DJ-1/ATP5F1B colocalization (*n* = 3 from 3 different differentiations). One-way ANOVA with post hoc Tukey’s test. *P < 0.05 compared with DMSO-treated cells without dox. ^#^*P* < 0.05 compared with DMSO-treated cells with dox. ^§^*P* < 0.005 compared with DMSO-treated cells without dox. ^&^*P* < 0.005 compared with DMSO-treated cells with dox. Error bars represent SEM. Scale bars, 10 µm.

We then assessed translocation of endogenous parkin to depolarized mitochondria in dopaminergic neurons. In basal conditions, endogenous parkin in dopaminergic neurons was essentially undetectable with immunofluorescence (Fig. 6a). However, after valinomycin treatment, endogenous parkin puncta became visible on mitochondria, probably due to concentration of the protein (Fig. 6a). These valinomycin-induced parkin puncta did not appear in iPSC-derived dopaminergic neurons from a PD patient with compound heterozygous *PRKN* mutations (deletion of exon 2 and duplication of exon 6)^17^ (Supplementary Fig. 4), confirming the specificity of the signal. We determined the area of colocalization of parkin with mitochondria per dopaminergic neuron, but found no difference between neurons with and without α-synuclein upregulation (Fig. 6b). In addition, levels of mitofusin 2 and MIRO1, two OMM proteins that are ubiquitinated by parkin upon mitochondrial depolarization and then degraded by the proteasome^31^, were similar between neurons with and without α-synuclein upregulation, both in basal conditions and after valinomycin treatment (Fig. 6c, d, f, g). Moreover, the amount of ubiquitinated endogenous mitofusin 2 after valinomycin treatment did not differ between neurons with and without α-synuclein upregulation (Fig. 6c, e).

Finally, valinomycin-induced translocation of endogenous DJ-1 to mitochondria^17^ was not affected by α-synuclein upregulation (Fig. 6i, j). Thus, PINK1, parkin, and DJ-1 appeared to function normally in the mitophagy pathway despite increased α-synuclein abundance.

### Actin depolymerization rescues mitophagy in human neurons with elevated α-synuclein

**Fig. 7:**
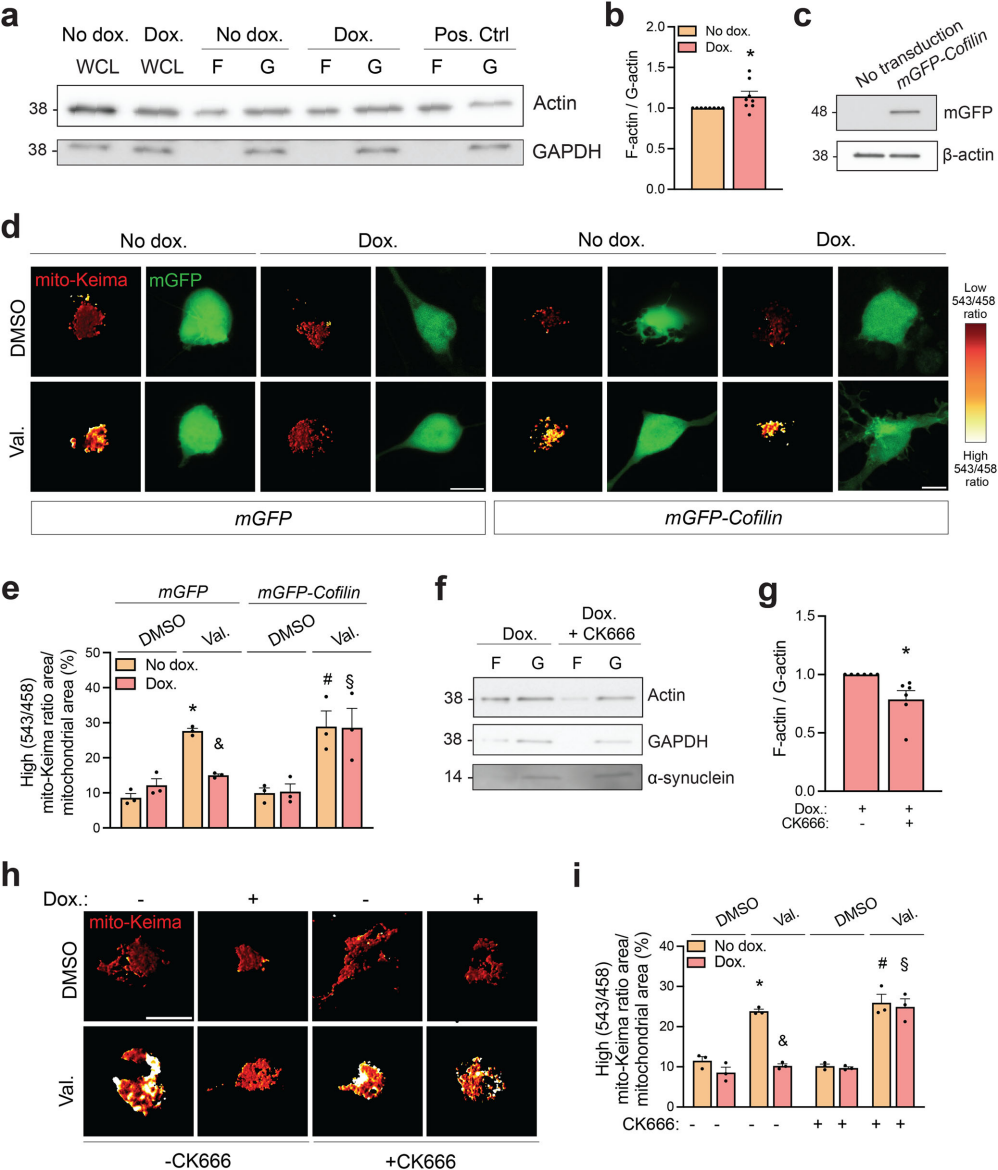
Actin depolymerization rescues mitophagy in iPSC-derived dopaminergic neurons with increased α-synuclein. **a** iPSC-derived neurons carrying the doxycycline (dox.)-inducible extra *SNCA* copy with or without dox. treatment (3 μg/mL, 5 days) were lysed, followed by ultracentrifugation to separate F-actin (F) and G-actin (G) fractions and western blotting. As a positive control (Pos. Ctrl.) for the assay, a phalloidin-containing F-actin enhancing solution was added to the ‘No dox.’ lysate to drive actin polymerization before ultracentrifugation. WCL, whole-cell lysate. **b** Quantification of F-actin/G-actin ratios, normalized to the No dox. condition (*n* = 8 from 8 different differentiations). Mann−Whitney Rank Sum test. *P < 0.05 compared with No dox. **c** Western blot of iPSC-derived neurons transduced with *mGFP-Cofilin*. **d** iPSC-derived neurons carrying the dox.-inducible *SNCA* copy were transduced with mito-Keima lentivirus in combination with lentivirus expressing either mGFP or mGFP-Cofilin. Dox. or vehicle treatment for 3 days was followed by 24 h exposure to DMSO or Val. before live ratiometric mito-Keima imaging. **e** Quantification of mitophagic flux (*n* = 3–4 experiments, each from a different differentiation, with at least 15 cells analyzed per condition per experiment). One-way ANOVA with post hoc Tukey’s test. *P < 0.01 compared to *mGFP*-transduced cells treated with DMSO without dox. ^#^*P* < 0.01 compared to *mGFP-Cofilin-*transduced cells treated with DMSO without dox. ^&^*P* < 0.05 compared to *mGFP-Cofilin-*transduced cells treated with Val. and dox. and compared to *mGFP-*transduced cells treated with Val. without dox. ^§^*P* < 0.01 compared to *mGFP-Cofilin-*transduced cells treated with DMSO and dox. **f** iPSC-derived neurons carrying the doxycycline (dox.)-inducible extra *SNCA* copy were treated with dox. (3 μg/mL, 5 days) without or with CK666 (100 μM, during the last 24 h of dox.) and lysed, followed by separation of F- and G-actin fractions and western blotting. **g** Quantification of F-actin/G-actin ratios, normalized to the dox. without CK666 condition (*n* = 6 from 6 different differentiations). Mann−Whitney Rank Sum test. *P < 0.005 compared to dox. without CK666. **h** iPSC-derived neurons carrying the dox.-inducible *SNCA* copy were transduced with mito-Keima lentivirus and treated for 3 days with dox. or vehicle. Cells were then pre-treated for 1 h with DMSO or CK666 (100 μM), followed by treatment for 24 h with DMSO or Val. alone or in combination with CK666 (100 μM) before live ratiometric imaging. **i** Quantification of (**h**) (*n* = 3 experiments from 3 different differentiations, with at least 15 cells analyzed per condition per experiment). One-way ANOVA with post hoc Tukey’s test. *P < 0.0001 compared to DMSO-treated cells without dox. or CK666. ^&^*P* < 0.0001 compared to cells treated with Val., dox. and CK666 and compared to cells treated with Val. without Dox. and without CK666. ^#^*P* < 0.0001 compared to DMSO-treated cells without dox. and with CK666. ^§^*P* < 0.0001 compared to cells treated with DMSO, dox. and CK666. Error bars represent SEM. Scale bars, 10 µm.

Recent work has shown that α-synuclein expression causes excessive stabilization of actin filaments and impaired mitophagy in *Drosophila* brain^32,33^. To determine the ratio of polymerized actin (F-actin) to monomeric actin (G-actin), iPSC-derived neurons were lysed in a buffer that maintains the G- and F-forms of actin, followed by ultracentrifugation of the lysates to pellet F-actin and western blot to measure actin levels in the pellet and supernatant fractions^32^. There was a small but significant increase in F-actin/G-actin ratio after upregulation of α-synuclein (Fig. 7a, b).

Transgenic overexpression of the actin-depolymerizing protein cofilin has been shown to rescue the mitophagy defect of α-synuclein-expressing flies^33^. We asked if interference with actin polymerization could also rescue the mitophagy defect induced by increased α-synuclein in human neurons. Interestingly, transduction of cofilin fully restored valinomycin-induced mitophagy in neurons with upregulated α-synuclein, while this had no effect on mitophagy in neurons with normal α-synuclein levels (Fig. 7c–e). We also tested the effect of CK666, a cell-permeable small molecule inhibitor of the actin-polymerizing Arp2/3 complex^34^ that has been implicated in the excessive actin filament stabilization in α-synuclein-expressing flies^33^. Importantly, CK666 reduced the F-actin/G-actin ratio in the doxycycline-treated cultures, while having no effect on α-synuclein levels (Fig. 7f, g), and rescued mitophagy in the doxycycline-treated neurons (Fig. 7h, i).

Taken together, the data suggested that depolymerization of the actin filament network reversed the mitophagy defect caused by elevated α-synuclein in human neurons.

## Discussion

The mechanisms by which increased α-synuclein abundance induces neurodegeneration are incompletely understood, but probably involve a combination of multiple pathways. Mitochondria have been reported to be one of the main targets of α-synuclein-induced damage^7–12^. Here, we show in *Drosophila* and human cells that elevated α-synuclein potently suppresses the cell’s ability to clear damaged mitochondria via mitophagy, without causing a general autophagy block.

To assess the effect of α-synuclein on mitophagy in vivo, we used a well-characterized mito-Keima-expressing *Drosophila* model^19^. Parkin-mediated mitophagy was robustly detected in flight muscle of adult wild-type mito-Keima flies, as previously reported^19^, but was drastically impaired by expression of wild-type or PD-linked mutant human α-synuclein. The magnitude of the mitophagy defect was similar in flies expressing wild-type and mutant α-synuclein, in line with previous studies that found no major difference in toxic effects of wild-type versus mutant human α-synuclein in *Drosophila*^35,36^. We generated a novel fly model that transgenically expresses the non-mitochondrial autophagy reporter Keima. Imaging in α-synuclein-expressing mito-Keima and Keima flies allowed us to directly compare the impact of α-synuclein on autophagic flux of mitochondrial versus non-mitochondrial cargo and revealed that non-mitochondrial autophagic flux was preserved despite the mitophagy defect. This suggested that α-synuclein did not interfere with the most downstream steps in the autophagy pathway that are likely independent of cargo type, such as autophagosome-lysosome fusion or lysosomal acidification. Interestingly, a recent study using the same mito-Keima fly model found that transgenic expression of human wild-type α-synuclein inhibited mitophagic flux in fly brain neurons^33^. In the same study, overall autophagic flux, as measured using the GFP-mCherry-Atg8a reporter, was diminished as well in α-synuclein transgenic fly neurons^33^. However, it should be noted that the GFP-mCherry-Atg8a reporter binds to membranes of all autophagosomes, irrespective of their cargo, and thus also labels mitochondria-containing autophagosomes. Therefore, if mitophagy is responsible for a major proportion of all autophagic events in a particular cell type, a selective, severe mitophagy block may also be detected as a decrease in general autophagic flux when assessed with GFP-mCherry-Atg8a imaging. By contrast, our comparison of mito-Keima and Keima flies allowed us to disentangle mitochondrial from non-mitochondrial autophagic flux, revealing a more selective deleterious impact of α-synuclein on mitophagy.

A limitation of *Drosophila* as a model organism for PD is that flies do not express an endogenous α-synuclein homolog. We therefore also assessed the effect of α-synuclein on mitophagy in human cells. We found that overexpression of wild-type α-synuclein, but not β-synuclein, disrupted valinomycin-induced mitophagic flux in human fibroblasts without affecting starvation-induced autophagy of non-mitochondrial cargo. However, as endogenous α-synuclein levels in human fibroblasts are extremely low and typically not detectable by western blotting^24,25^, overexpression of α-synuclein in these cells should still be considered as ectopic. We therefore engineered a more pathophysiologically relevant model that allows titratable upregulation of α-synuclein in human iPSC-derived neurons. We show that a limited, approximately twofold increase in α-synuclein, similar to the increase in PD patients with an *SNCA* triplication, was already sufficient to largely abolish mitophagic flux. To our knowledge, this is the first demonstration of a deleterious effect of elevated wild-type α-synuclein levels on mitophagy in an isogenically controlled human neuron model. As in *Drosophila* and human fibroblasts, Keima imaging showed no effect of elevated α-synuclein on non-mitochondrial autophagic flux. It should be kept in mind, however, that the α-synuclein upregulation in our experiments in iPSC-derived neurons was relatively acute. It is possible that longer exposure to increased α-synuclein, as in *SNCA* triplication neurons, would eventually also impair non-mitochondrial autophagy, given previous reports on deleterious effects of α-synuclein overexpression or aggregates on autophagosome transport and autophagosome-lysosome fusion^37,38^. Another limitation of our Keima experiments in iPSC-derived neurons was that we measured non-mitochondrial autophagy only in basal conditions because we previously found that amino acid starvation and Torin1, classical inducers of non-selective autophagy in many tissues, were not effective at upregulating autophagy in these cells^17^. Effects of increased α-synuclein levels on non-mitochondrial autophagy in iPSC-derived neurons might potentially become more obvious under conditions that induce non-selective autophagy more strongly.

PINK1 accumulation and activation (as measured by phospho-ubiquitin formation) and parkin translocation and activation (as assessed by ubiquitination and degradation of mitofusin 2 and degradation of MIRO1) and DJ-1 translocation to mitochondria after mitochondrial depolarization were all intact in neurons with upregulated α-synuclein, suggesting that α-synuclein did not interfere with the function of these three mitophagy-promoting proteins linked to autosomal recessive PD. Recently, Hou et al. also found no reduction of phospho-ubiquitin formation upon mitochondrial damage in cells with increased wild-type α-synuclein^39^. Shaltouki et al. reported that basal MIRO1 abundance was increased in iPSC-derived *SNCA* triplication neurons^40^, but we could not confirm this in our iPSC-derived neurons with upregulated α-synuclein, possibly due to the relatively short duration of α-synuclein upregulation in our system.

Ordonez et al. first reported that α-synuclein expression in fly brains dysregulates actin dynamics, leading to excessive F-actin stabilization^32^. Here, we found a similar increase in F-actin/G-actin ratio in human neurons with upregulated α-synuclein. The observed increase in F-actin/G-actin ratio was small, but it should be kept in mind that these measurements were made in whole-cell lysates. If the effect of α-synuclein on actin dynamics is more pronounced in specific subcellular sites, e.g., around mitochondria, this effect could get diluted in whole-cell lysates. Recent evidence from non-neuronal cell lines indicates that F-actin can assemble locally around acutely or chronically damaged mitochondria^41–43^. Perimitochondrial actin dynamics play specific roles in the initial stages of parkin-mediated mitophagy, such as disassembly of aggregated damaged mitochondria into smaller pieces to facilitate mitophagic engulfment^44^ and encapsulation of parkin-positive damaged mitochondria to prevent refusion with healthy mitochondria^42^. We hypothesize that elevated α-synuclein may locally disrupt the perimitochondrial actin dynamics that are necessary for efficient PINK/parkin-mediated mitophagy. Remarkably, we found that treatment with CK666, an inhibitor of the actin-polymerizing Arp2/3 complex, or overexpression of the actin-severing protein cofilin restored mitophagy in human neurons with upregulated α-synuclein, consistent with the rescuing effect of genetic F-actin destabilization on the mitophagy defect of α-synuclein transgenic flies^33^. Interestingly, the Arp2/3 complex has been shown to mediate local actin polymerization around damaged mitochondria, as this is blocked by CK666^42,43^. Cofilin may also act locally around mitochondria, as it translocates to the OMM of dysfunctional mitochondria and colocalizes with actin on the OMM^41,45,46^. Super-resolution microscopy will be needed to analyze the effects of α-synuclein on Arp2/3- and cofilin-regulated perimitochondrial dynamics of endogenous actin in neurons.

Our study has several limitations. First, we used only one iPSC clone for differentiation of neurons with a doxycycline-inducible extra *SNCA* copy. A second limitation of our neuronal inducible α-synuclein expression system was the use of doxycycline. Doxycycline has been reported to impair mitochondrial function^47,48^ and inhibit α-synuclein aggregation^49^. However, we showed that doxycycline had no effect on neuronal mitophagy in the absence of the *SNCA* transgene. Moreover, we also found an inhibitory effect of elevated α-synuclein on mitophagic flux in our *Drosophila* and fibroblast models, where no doxycycline was applied. Another limitation was that we did not address the molecular mechanism by which elevated α-synuclein levels lead to F-actin stabilization. This could be mediated via previously identified interactions of α-synuclein with spectrin^32^ and numerous other components of the actin cytoskeleton^50^.

A recent study revealed that Lewy bodies and Lewy neurites in PD brains, in addition to α-synuclein, also contain numerous distorted, clustered mitochondria and structures reminiscent of autophagosomes and lysosomes^18^. Our finding that increased α-synuclein abundance paralyzes the cell’s ability to deliver damaged mitochondria to the lysosomal lumen may help understand how Lewy pathology originates in the pathogenesis of PD.

## Methods

### Antibodies

The following primary antibodies were used for western blot (WB) or immunofluorescence (IF): mouse anti-α-synuclein (WB, 1:500; BD Biosciences 610787), mouse anti-β-actin (WB, 1:5000; Sigma, A5441), rabbit anti-GAPDH (WB, 1:2000; Invitrogen, PA1-16777), rabbit anti-HSP60 (WB, 1:1000; IF, 1:1000; Abcam, ab53109), mouse anti-ATP5F1B (WB, 1:1000, IF, 1:500; Abcam, ab14730), mouse anti-mitofusin 2 (WB, 1:1000; Abcam, ab56889), rabbit anti-TH (WB, 1:500; IF, 1:500; Sigma, AB152), sheep anti-TH (IF, 1:300; Thermo Fisher, PA1-4679), mouse anti-MAP2 (IF, 1:500; Sigma, M1406), rabbit anti-PINK1 (WB, 1:1000; Novus Biologicals, BC100-494), rabbit anti-phospho-ubiquitin^S65^ (IF, 1:250; Sigma, ABS1513-I), mouse anti-parkin (IF, 1:250; EMD Millipore, 05-882), rabbit anti-DJ-1 (IF, 1/250; Abcam, ab18257), mouse anti-RHOT1 (anti-MIRO1) (WB, 1:500; Abnova, H000055288-M01), mouse anti-FLAG (IF, 1:500; Sigma, F3165), rabbit anti-FLAG (WB, 1:1000; Sigma, F7425), rabbit anti-TurboGFP (WB, 1:2000; Origene, TA150071), rabbit anti-mGFP (WB, 1:5000; Origene, TA150122), rabbit anti-OCT4 (IF, 1:100; Santa Cruz, sc-9081), rabbit anti-NANOG (IF, 1:300; Thermo Fisher, PA1-097X), mouse anti-TRA-1-60 (IF, 1:200; Cell Signaling Technology, 4746), mouse anti-TRA-1-81 (IF, 1:200; Cell Signaling Technology, 4745), rabbit anti-Sox2 (IF, 1:500; Merck Millipore, AB5603) and mouse anti-SSEA4 (IF, 1:200; Santa Cruz, sc-21704). Mouse anti-pan actin antibody (WB, 1:1000; Cytoskeleton, AAN02-S) was used for F-actin/G-actin ratio determination as part of the G-actin/F-actin In Vivo Assay Kit (Cytoskeleton, BK037; cfr. infra). Secondary antibodies for WB were peroxidase-linked anti-mouse (SAB3700934, Sigma) and anti-rabbit (SAB3701095, Sigma). Secondary antibodies for IF were donkey Alexa Fluor anti-mouse 488 and 555 (Thermo Fisher, a21202, a21206), anti-rabbit 488 and 555 (Thermo Fisher, a31570, a31572) and anti-sheep 647 (Thermo Fisher, a21448), goat Alexa Fluor anti-rabbit 488 (Thermo Fisher, a11034), anti-mouse (IgM) 555 (Thermo Fisher, a21426) and anti-mouse (IgG) 555 (Thermo Fisher, a21424).

### cDNAs and lentiviral production

pCMV6-Entry vectors encoding FLAG-tagged human α-synuclein (RC210606) and β-synuclein (RC215165), pCMV6-AC-GFP vectors encoding TurboGFP-tagged human α- (RG210606) and β-synuclein (RG215165), pLenti-C-mGFP-P2A-Puro lentiviral control particles (PS100093V) and pLenti-C-mGFP-P2A-Puro encoding mGFP-tagged human cofilin (RC203585L4V) were from Origene. The mito-Keima construct (mt/mKeima/pIND(SP1)) was a gift from Dr. A. Miyawaki (RIKEN Brain Science Institute, Japan). The Keima construct (mKeima-Red-N1) was a gift from Dr M. Davidson (Addgene, 54597). The pEGFP-C1 vector was a gift from Dr P. Vangheluwe (KU Leuven). Cloning of Keima and mito-Keima cDNA into lentivirus and production of lentiviral particles were described before^17^.

### Drosophila genetics

All *Drosophila melanogaster* crosses were kept on standard corn meal and molasses food at 25 °C with a 12-h day-night cycle. New wild-type and mutant human *SNCA*-expressing *Drosophila* models were generated with codon-optimized, synthesized *SNCA* coding sequences (IDT, Supplementary Table 1), which were inserted with Gibson assembly into EcoRI- and XbaI-digested pUASTattB plasmid^51^. Keima cDNA was cloned with Gibson Assembly into EcoRI and Xho1 sites. Plasmids encoding Keima or α-synuclein were inserted into chromosome 3 R integration site VK20 or 3 L VK05 attP docking site, respectively, for phiC31-mediated transformation by Bestgene. Resulting flies were backcrossed for 5 generations into the same *w*^-^ Canton-S (CS) reference strain that was used for the previously generated UAS-smGdP::V5 line (integration of an empty pUASTattB backbone into VK05)^52^. *Drosophila* expressing mito-Keima were previously described^19^ and backcrossed for 10 generations into the *w*^*-*^ CS reference strain. Parkin TRiP RNAi, control TRiP RNAi, and Atg1^K38A^ were obtained from Bloomington Stock Center (Indiana, USA; RRID: BDSC_37509, BDSC_31603, BDSC_60736).

### Human skin fibroblasts

Fibroblasts from a 57-year-old healthy female were cultured and transiently transfected with 3 µg cDNA using the Neon Transfection System (Invitrogen, MPK1096) according to the manufacturer’s instructions, as described^17,53^. Transfection efficiency was 44.2 ± 0.8% (*n* = 3) for mito-Keima, 46.9 ± 2.2% (*n* = 3) for Keima, 17.7 ± 1.0% (*n* = 3) for *SNCA-TurboGFP*, 23.6 ± 7.1% (*n* = 3) for *SNCB-TurboGFP*, 13.4 ± 2.0% (*n* = 3) for *SNCA-FLAG* and 21.3 ± 4.1% (*n* = 3) for *SNCB-FLAG*. All procedures were approved by the Ethical Committee UZ/KU Leuven and performed in accordance with the World Medical Association Declaration of Helsinki. Written informed consent was obtained from all cell donors. Regular testing confirmed absence of *Mycoplasma*. Experiments were performed at passage numbers 5–14.

### Generation of inducible α-synuclein iPSC line

An extra copy of wild-type human *SNCA*, regulated by a doxycycline-inducible promotor, was inserted into a control iPSC line (Sigma, iPSC0028) as follows. Using the method originally described by ref. ^54^, we previously inserted an FRT-flanked donor cassette for doxycycline-inducible expression into the *AAVS1* locus of the iPSC0028 line, as described in detail in ref. ^55^. All quality controls on the resulting iPSC line were previously reported: 3′ and 5′ junction assay PCR to demonstrate correct insertion, southern blot to demonstrate absence of random inserts, pluripotency assays, SNP profiling to demonstrate cell identity, and aCGH to demonstrate genome integrity^55^. To create the inducible *SNCA* line, we used recombinase-mediated cassette exchange, as described^54^. A schematic of the procedure and the plasmids is shown in Supplementary Fig. 5. The human *SNCA* coding sequence (IDT) was: CTCGTTTAGTGAACCGTCAGATCGCTTAAGGCCACCATGGATGTATTCATGAAAGGACTTTCAAAGGCCAAGGAGGGAGTTGTGGCTGCTGCTGAGAAAACCAAACAGGGTGTGGCAGAAGCAGCAGGAAAGACAAAAGAGGGTGTTCTCTATGTAGGCTCCAAAACCAAGGAGGGAGTGGTGCATGGTGTGGCAACAGTGGCTGAGAAGACCAAAGAGCAAGTGACAAATGTTGGAGGAGCAGTGGTGACGGGTGTGACAGCAGTAGCCCAGAAGACAGTGGAGGGAGCAGGGAGCATTGCAGCAGCCACTGGCTTTGTCAAAAAGGACCAGTTGGGCAAGAATGAAGAAGGAGCCCCACAGGAAGGAATTCTGGAAGATATGCCTGTGGATCCTGACAATGAGGCTTATGAAATGCCTTCTGAGGAAGGGTATCAAGACTACGAACCTGAAGCCTAAACGCGTGGGGGAGGCTAACTGAAACACGGAA. The donor plasmid was flanked at both sides with 2 cHS4 insulator cassettes to inhibit silencing, as described^54^. After nucleofection of the donor plasmid together with an FLPase plasmid (using the Amaxa nucleoporator, program 16, hESC nucleofection kit) as described^54^, positive selection with puromycin (120–300 ng/µl; Sigma) followed by negative selection with 0.5 µM fialuridine (Sigma, SML0632) were used to select for correctly recombined colonies. Correct integration of the cassette was demonstrated by 3′ and 5′ junction assay PCR.

The iPSC line from the PD patient with compound heterozygous *PRKN* mutations (deletion of exon 2 and duplication of exon 6) has been characterized previously^17^.

### Quality controls for inducible α-synuclein iPSC line

The inducible α-synuclein iPSC line successfully underwent quality controls for pluripotency, cell identity, and genome integrity (Supplementary Fig. 6). For IF staining for pluripotency markers (Supplementary Fig. 6a), iPSCs were seeded on glass coverslips coated with matrigel (VWR, BDAA356277) in Essential 8 flex medium (Thermo Fisher). Once colonies were formed, cells were fixed with 4% paraformaldehyde for 15 min. Cells were permeabilized with 0.1% Triton X-100 in PBS for 15 min, blocked with 5% goat serum (Dako, X0907) for 30 min, and immunostained overnight with primary antibodies.

For analysis of trilineage differentiation potential, iPSCs were subjected to spontaneous differentiation mediated by the formation of embryoid bodies (EBs) and subsequently analyzed for trilineage differentiation using ScoreCard methodology (Thermo Fisher) (Supplementary Fig. 6b). On the day of EB formation, 60–80% confluent iPSCs were washed with PBS and then incubated with 0.5 mM EDTA for 1–3 min to dissociate colonies. Cells were harvested in Essential 8 flex medium and spun down at 300 g for 5 min. 500,000 cells were stored for subsequent RNA extraction (day 0 sample). 15 × 10^6^ cells/1.5 ml were seeded into 24-well Corning Ultra-Low Attachment Surface plates (Corning). Every other day, half of the medium was replaced by Essential 6 medium (Thermo Fisher). On day 7, cells were harvested. RNA was extracted from the day 0 and day 7 samples with the GenElute Mammalian Total RNA Miniprep kit (Sigma). cDNA synthesis from extracted RNA was performed with the Superscript III kit (Thermo Fisher), followed by qPCR according to manufacturer’s protocol with TaqMan hPSC Scorecard Panel (Thermo Fisher). Data were analyzed with hPSC Scorecard software (online tool Thermo Fisher).

For analysis of genomic identity by DNA fingerprinting, a reference set of 32 single nucleotide polymorphisms (SNPs) was analyzed with qPCR using TaqMan SNP Genotyping assays (Thermo Fisher) and TaqMan Genotyper software in the undifferentiated original control iPSC line (iPSC0028) and the undifferentiated iPSC0028 line in which the doxycycline-inducible extra *SNCA* copy had been introduced (Supplementary Fig. 6c).

Karyotype analysis using array comparative genomic hybridization (aCGH) was performed with the CytoSure Syndrome Plus 60 K array (Oxford Gene Technology, UK), a platform that has genome-wide coverage with enrichment of target regions. Details of the array designs are available from Oxford Gene Technology (http://www.ogt.co.uk/) or the authors. Genomic DNA was labeled for 4 h or overnight using the CytoSure Labelling Kit (Oxford Gene Technology) without enzyme digestion. Hybridization was performed from 24 to 60 h in a rotator oven (SciGene, USA) at 65 °C. Washing of arrays was performed using wash solutions (Agilent Technologies) with a Little Dipper Microarray Processor (SciGene). Arrays were scanned using an Agilent microarray scanner at 2-μm resolution, followed by calculation of signal intensities using Feature Extraction software (Agilent Technologies). Visualizations of results and data analysis were performed using the CytoSure Interpret Software (Oxford Gene Technology) and the circular binary segmentation algorithm. One sample was hybridized twice in dye-swap experiments, labeled with Cy5 and Cy3 and hybridized versus Cy3- and Cy5-labeled reference DNA, respectively. The dye swap increases the sensitivity which in turn allows a more accurate detection of smaller imbalances, refinement of the breakpoint, and mosaicism. Quality control metrics were monitored with CytoSure Interpret software (Oxford Gene Technology). Genomic coordinates were based on build hg19. No significant genome-wide aberrations were found.

### Differentiation of iPSCs to dopaminergic neurons

iPSCs were expanded until confluent on matrigel (VWR, BDAA356277)-coated 6-well plates (VWR, 734-2323) in mTeSR basal medium supplemented with mTeSR 5× supplement (Stemcell Technologies, 85850), penicillin (10 µ/ml; Thermo Fisher, 15140112) and streptomycin (10 µg/ml; Thermo Fisher, 15140112). For splitting, cells were washed briefly with PBS, incubated with ReLeSR (Stemcell Technologies, 5873) at room temperature for 4 min, collected in mTeSR medium, and seeded. iPSCs were differentiated to dopaminergic neurons as described^17,56^. To dissociate cells, iPSCs were incubated with 1 ml/well accutase (400–600 µ/ml, Sigma, A6964) for 4 min at 37 °C. Cells were suspended in accutase and the plate was rinsed with 2 ml/well of mTeSR to collect remaining cells. After centrifugation for 5 min at 300 g, supernatant was discarded and cells were resuspended in mTeSR containing 2 µl/ml Y-27632 ROCK inhibitor (Sigma, 688001) and seeded on matrigel-coated 6-well plates. To induce neuronal differentiation, medium was switched on the next day (day 0 of induction) to KSR (KnockOut DMEM medium [Life Technologies, 10829018] containing 15% KnockOut Serum Replacement [Life Technologies, 10828010], 2 mM L-glutamine, 10 mM β-mercaptoethanol, 1% non-essential amino acids, 10 µ/ml penicillin and 10 µg/ml streptomycin) supplemented with LDN-193189 (100 nM, Miltenyi Biotec, 130-106-540) and SB-431542 (10 µM, R&D systems, 1614). Medium was replaced on day 1 by KSR supplemented with LDN-193189 (100 nM), SB-431542 (10 µM), FGF8b (100 ng/ml, R&D systems, 423-F8), SHH (C25II) (100 ng/ml, R&D systems, 464-SH) and purmorphamine (2 µM, Merck Millipore, 540223). On day 3, CHIR-99021 (3 µM, Tocris, 4423) was added. From day 5 onwards KSR medium was gradually shifted (¼ NB + ¾ KSR on day 5, ½ NB + ½ KSR on day 7, ¾ NB + ¼ KSR on day 9) to NB medium (Neurobasal medium [Life Technologies, 21103049] supplemented with B27 [Life Technologies,12587010] and N2 [Life Technologies, 17502048], 2 mM L-Glutamine, 10 µ/ml penicillin and 10 µg/ml streptomycin). On day 11, medium was completely replaced by NB medium containing CHIR-99021 (3 µM), BDNF (20 ng/ml, Peprotech, AF-450-02), ascorbic acid (0.2 mM, A4544, Sigma), GDNF (20 ng/ml, Peprotech, AF-450-10), TGFβ3 (1 ng/ml, Peprotech, 100-36E), dibutyryl cAMP (0.5 mM, Sigma, D0627) and DAPT (10 µM, Tocris, 2634). On day 13, cells were dissociated using accutase and replated 1:1 on matrigel-coated plates in differentiation medium (same composition as day 11 NB medium but without CHIR-99021) and medium was replaced every other day. On day 20, cells were dissociated again using accutase and replated at high cell density (±150,000 cells/well for a 12-well plate, ±350,000 cells/well for a 6-well plate) on plates pre-coated with poly-L-ornithine (50 µg/ml, Sigma, P3655) and laminin (10 µg/ml, Sigma, L2020) and maintained in differentiation medium until day 50 by replacing medium three times a week. Once a week the fresh medium contained 10 µg/ml laminin.

Transcription of the extra *SNCA* copy in neurons derived from the inducible α-synuclein iPSC line was induced by incubating cells in fresh growth medium containing 3 μg/mL doxycycline (Sigma, D9891). This concentration was selected based on previous titration experiments^54^ and was also used in previous studies where iPSCs with a doxycycline-inducible cassette incorporated into the *AAVS1* locus were differentiated to other neuronal cell types, such as nociceptor neurons^57^ and cortical neurons^58^.

Neurons were transduced with lentivirus as described^17^. Transduction efficiency in neurons was 19.9 ± 2.1% (*n* = 3) for mito-Keima and 17.3 ± 3.3% (*n* = 3) for Keima.

### Mito-Keima and Keima imaging

Live ratiometric mito-Keima imaging was performed as described in *Drosophila* flight muscle^19^, fibroblasts^17,53^, and iPSC-derived neurons^17^, and Keima imaging was done analogously. Cells were analyzed using Leica TCS SP5 II or SP8 confocal microscopes equipped with a 63× objective lens (HCPL APO 63×/1.4 CS2), a multi-argon laser (458, 476, 488) and a He/Ne laser (543). Keima and mito-Keima were imaged in 2 channels via 2 sequential excitations (458 nm, green; 543 nm, red) and using a 600–695 nm emission range. Images from random microscopic fields were captured and analyzed by an investigator blinded to the experimental condition. For fly muscle analysis, at least 10 z-stacks were taken per fly. Ratio (543/458) images were created using the Ratio Plus plugin in ImageJ. High (543/458) mito-Keima or Keima ratio areas were segmented and quantified with the Analyze Particles plugin in ImageJ. The parameter high mito-Keima (543/458) ratio area/total mitochondrial area was used as an index of mitophagy. Total mitochondrial area was determined by segmenting the area of total emission at 458 nm excitation in the original image (example shown in Supplementary Fig. 7), which was also quantified using the Analyze Particles plugin. The parameter high Keima (543/458) ratio area/total cell area was used as an index of non-mitochondrial autophagy. Total cell area was quantified by measuring the total Keima emission area at 458 nm excitation (example shown in Supplementary Fig. 7) using the Analyze Particles plugin. For easy visualization of the level of mitophagy or autophagy in Figs. 3b, g, 4i, k, m, and 7d, h, a red hot lookup table (LUT) was applied to the ratio images in ImageJ. In some experiments, (mito-)Keima was imaged in cells that also expressed EGFP, TurboGFP or mGFP, as previous work showed that this can be done without significant cross-excitation, cross-detection, or resonance transfer^20^. For *SNCA* transfection and *cofilin* transduction for live imaging experiments, we used constructs tagged with GFP instead of the smaller FLAG tag in order to be able to identify transfected/transduced cells without immunostaining. LysoTracker (DND-26, Thermo Fisher) was imaged using a 488 nm excitation and a 495–550 nm emission filter^19^. Valinomycin was from Sigma (V3639), bafilomycin A1 from Abcam (ab120497), and CK666 from MedChemExpress (HY-16926).

### Immunocytochemistry, confocal microscopy, and western blot

Immunostaining of cultured cells and confocal imaging were performed as described^17,53^. TOTO-3 was from Thermo Fisher (T3604). Random confocal images were acquired and analyzed by an investigator blinded to genotype and experimental condition. To quantify colocalization of phospho-ubiquitin with ATP5F1B and of parkin with HSP60, the colocalization image creator plugin in ImageJ was used. Channels of z-stack images were segmented by applying an intensity threshold and a binary colocalization image was generated in which the colocalized areas in the z-layers are projected^59^. Subsequently, the area of colocalization in the binary colocalization image was measured using the Analyze Particles plugin in ImageJ and divided by total TH-positive area. Western blot was performed as described^17,53^. All blots were processed in parallel and derived from the same experiments.

### F-actin/G-actin ratio measurement

The G-Actin/F-Actin In Vivo Assay Kit (Cytoskeleton, BK037) was used to determine F-actin/G-actin ratios. iPSC-derived neurons (1–6 × 10^6^ cells) were homogenized in Lysis and F-actin Stabilization Buffer (100 μL buffer per 500.000 cells) supplemented with 1× Protease Inhibitor Cocktail and 1 mM ATP. Lysates were centrifuged at 350 × *g* for 5 min to pellet cell debris, followed by ultracentrifugation at 100.000 × *g* for 1 h at 37 °C to pellet F-actin. The pellet was solubilized by incubation in F-Actin Depolymerization Buffer for 1 h. Solubilized pellet (F-actin) and supernatant (G-actin) samples were immunoblotted. The positive control consisted of a phalloidin-containing F-actin Enhancing Solution (part of the kit) that is incubated with the lysate at 37 °C for 10 min to drive actin polymerization before ultracentrifugation.

### Statistics

Significance of differences was analyzed with two-tailed Student’s *t-*test for comparison between two groups with equal variances, with Mann–Whitney Rank Sum test for comparison between two groups with unequal variances, and with one-way ANOVA and post hoc Tukey’s test for comparison between more than two groups (GraphPad Prism 9, GraphPad Software, Inc.). Values represent mean ± standard error of the mean (SEM). A *P* < 0.05 was considered statistically significant.

### Reporting summary

Further information on research design is available in the Nature Research Reporting Summary linked to this article.

### Supplementary information

Supplementary Material

Reporting Summary

## Data Availability

The data analyzed during this study are available from the corresponding author upon request.
