# Supplementary material for: Elevated α-synuclein levels inhibit mitophagic flux
Source: NPJ Parkinsons Dis. 2024 Apr 9;10:80. doi: 10.1038/s41531-024-00696-0 (PMC11004019; doi:10.1038/s41531-024-00696-0)
Supplement: Supplementary file 1 — Supplementary Material [file 41531_2024_696_MOESM1_ESM.pdf]

Supplementary Table 1. Custom synthesized DNA fragments

SNCA (wild-type)	AACTCTGAATAGGGAATTGGGCAAACATGGACGTCTTTATGAAGGGACTGAGTA<br>AAGCGAAGGAGGGTGTGGTGGCGGCCGCAGAAAAAACGAAGCAGGGCGTGGCA<br>GAGGCGGCGGGAAAGACGAAGGAAGGAGTGCTGTATGTTGGCTCGAAAACCAAG<br>GAAGGCGTCGTGCACGGCGTTGCCACAGTTGCGGAGAAGACGAAGGAGCAGGTA<br>ACCAACGTAGGAGGAGCTGTAGTAACAGGCGTAACCGCCGTTGCCCAAAAGACC<br>GTCGAGGGAGCTGGTTCGATAGCCGCTGCTACCGGTTTTGTAAAAAAAGATCAGT<br>TGGGGAAAAACGAAGAGGGTGCTCCGCAGGAGGGAATCCTGGAAGACATGCCGG<br>TGGACCCAGATAATGAGGCATACGAGATGCCATCCGAAGAAGGCTACCAGGATT<br>ATGAACCAGAGGCATAAAGGATCTTTGTGAAGGAACCT
SNCA (A30P)	AACTCTGAATAGGGAATTGGGCAAACATGGACGTCTTTATGAAGGGACTGAGTA<br>AaGCGAAGGAGGGTGTGGTGGCGGCCGCAGAAAAAACGAAGCAGGGCGTGGCAG<br>AGGCGCCGGGAAAGACGAAGGAAGGAGTGCTGTATGTTGGCTCGAAAACCAAGG<br>AAGGCGTCGTGCACGGCGTTGCCACAGTTGCGGAGAAGACGAAGGAGCAGGTAA<br>CCAACGTAGGAGGAGCTGTAGTAACAGGCGTAACCGCCGTTGCCCAAAAGACCG<br>TCGAGGGAGCTGGTTCGATAGCCGCTGCTACCGGTTTTGTAAAAAAAGATCAGTT<br>GGGGAAAAACGAAGAGGGTGCTCCGCAGGAGGGAATCCTGGAAGACATGCCGGT<br>GGACCCAGATAATGAGGCATACGAGATGCCATCCGAAGAAGGCTACCAGGATTA<br>TGAACCAGAGGCATAAAGGATCTTTGTGAAGGAACCT
SNCA (E46K)	AACTCTGAATAGGGAATTGGGCAAACATGGACGTCTTTATGAAGGGACTGAGTA<br>AaGCGAAGGAGGGTGTGGTGGCGGCCGCAGAAAAAACGAAGCAGGGCGTGGCAG<br>AGGCGGCGGGAAAGACGAAGGAAGGAGTGCTGTATGTTGGCTCGAAAACCAAGA<br>AAGGCGTCGTGCACGGCGTTGCCACAGTTGCGGAGAAGACGAAGGAGCAGGTAA<br>CCAACGTAGGAGGAGCTGTAGTAACAGGCGTAACCGCCGTTGCCCAAAAGACCG<br>TCGAGGGAGCTGGTTCGATAGCCGCTGCTACCGGTTTTGTAAAAAAAGATCAGTT<br>GGGGAAAAACGAAGAGGGTGCTCCGCAGGAGGGAATCCTGGAAGACATGCCGGT<br>GGACCCAGATAATGAGGCATACGAGATGCCATCCGAAGAAGGCTACCAGGATTA<br>TGAACCAGAGGCATAAAGGATCTTTGTGAAGGAACCT
SNCA (A53T)	AACTCTGAATAGGGAATTGGGCAAACATGGACGTCTTTATGAAGGGACTGAGTA<br>AAGCGAAGGAGGGTGTGGTGGCGGCCGCAGAAAAAACGAAGCAGGGCGTGGCA<br>GAGGCGGCGGGAAAGACGAAGGAAGGAGTGCTGTATGTTGGCTCGAAAACCAAG<br>GAAGGCGTCGTGCACGGCGTTaCCACAGTTGCGGAGAAGACGAAGGAGCAGGTA<br>ACCAACGTAGGAGGAGCTGTAGTAACAGGCGTAACCGCCGTTGCCCAAAAGACC<br>GTCGAGGGAGCTGGTTCGATAGCCGCTGCTACCGGTTTTGTAAAAAAAGATCAGT<br>TGGGGAAAAACGAAGAGGGTGCTCCGCAGGAGGGAATCCTGGAAGACATGCCGG<br>TGGACCCAGATAATGAGGCATACGAGATGCCATCCGAAGAAGGCTACCAGGATT<br>ATGAACCAGAGGCATAAAGGATCTTTGTGAAGGAACCT

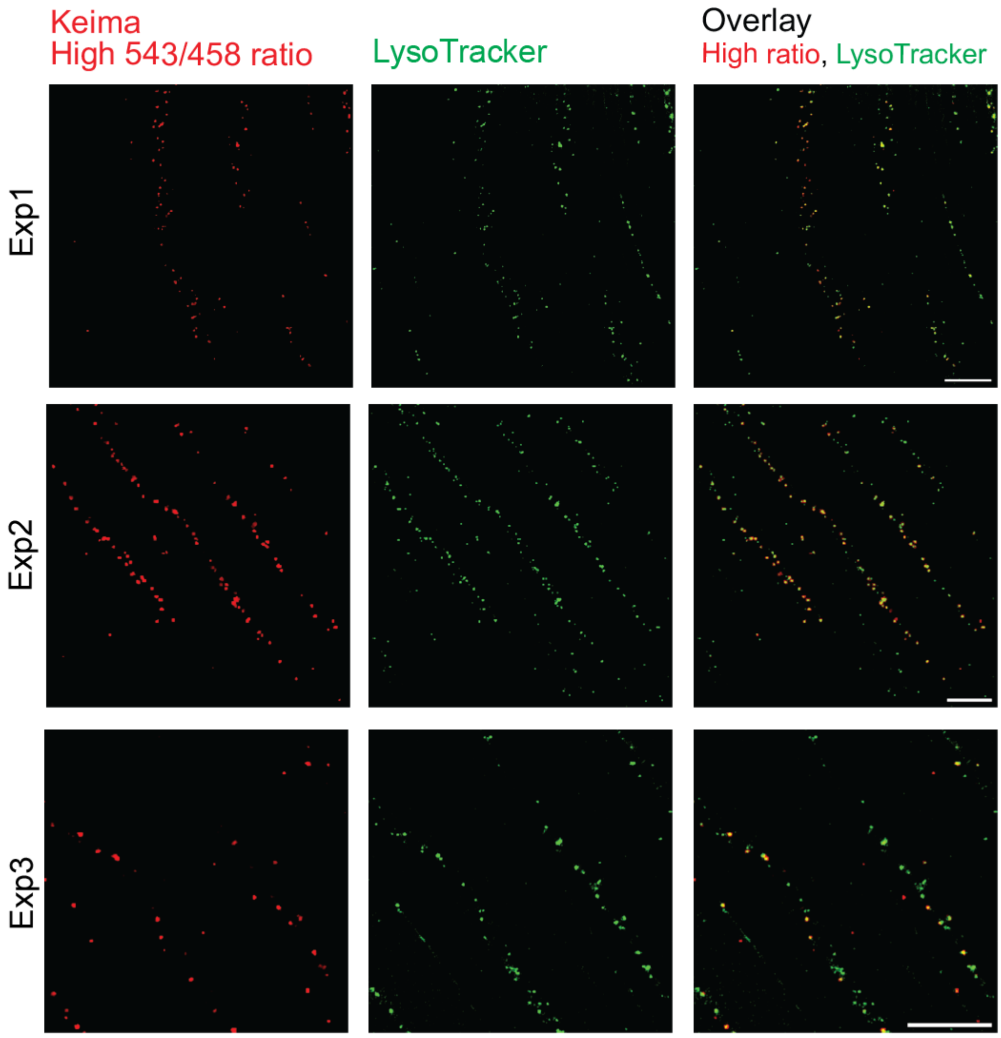

SUPPL. FIG. 1: Colocalization of high 543/458 ratio Keima puncta with lysosomes in *Drosophila* flight muscle. Additional examples of confocal images of Keima-expressing indirect flight muscle of 4-week-old CTRL1 flies (*w1118;;UAS-Keima,mef-2-GAL4/+)* from 3 different crosses (Exp1-3) labeled with Lysotracker (100 nM), showing that the vast majority of high 543/458 ratio ('acidic') Keima puncta colocalize with lysosomes. Scale bars, 10μm.

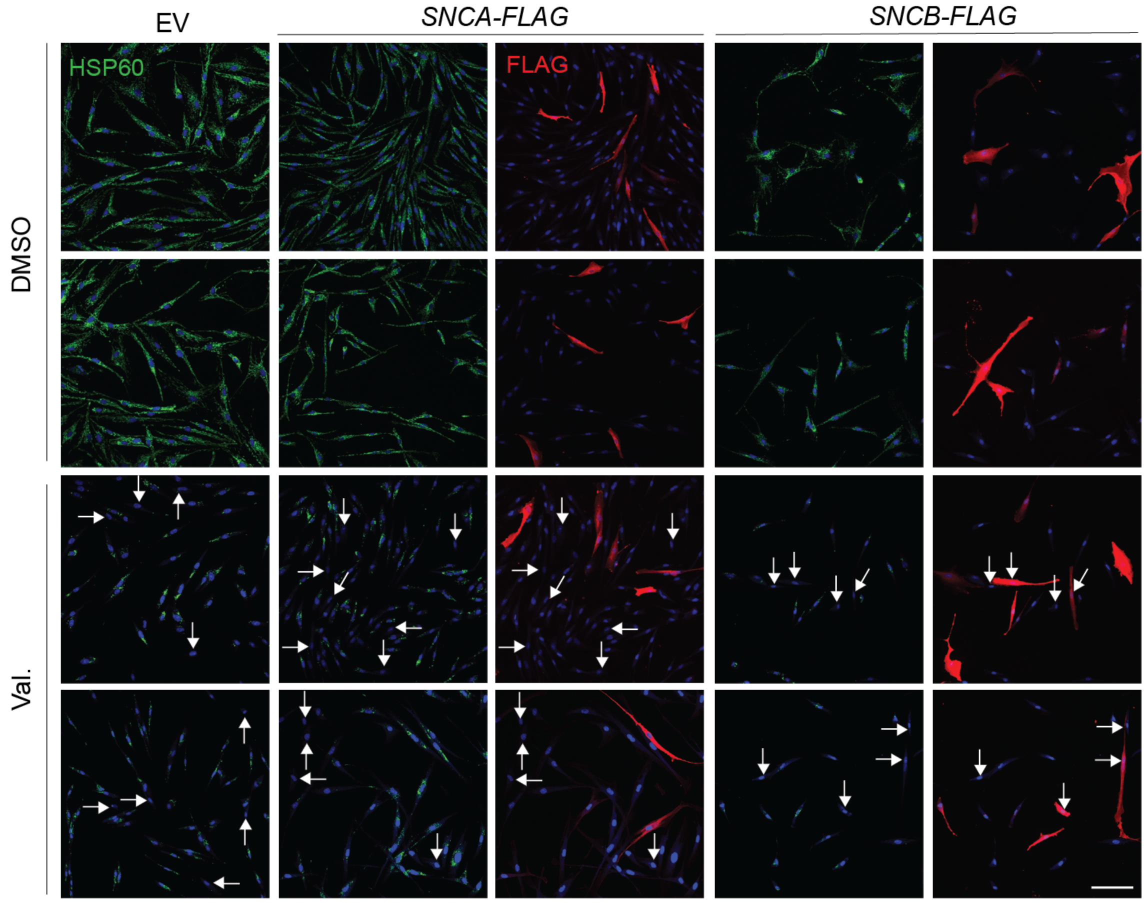

SUPPL. FIG. 2: Additional examples of effect of α-synuclein overexpression on mitophagy in fibroblasts as assessed by HSP60 immunocytochemistry. Control fibroblasts transfected with empty vector (EV), FLAG-tagged α-synuclein (*SNCA-FLAG*) or FLAG-tagged β-synuclein (*SNCB-FLAG*) were treated with DMSO or valinomycin (Val., 1 μM) for 48 h and immunostained for mitochondrial matrix protein HSP60. Nuclei were stained with TOTO-3 (blue). *Arrows* indicate examples of cells without detectable HSP60 staining. Scale bar, 20 μm.

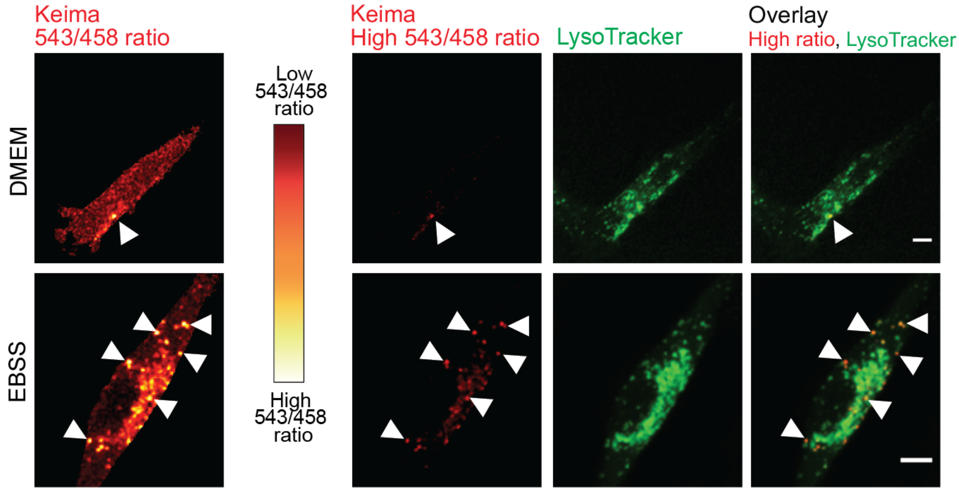

SUPPL. FIG. 3: Colocalization of high 543/458 ratio Keima puncta with lysosomes in fibroblasts. Human control fibroblasts were transfected with Keima. After 24 h, cells were incubated for 3 h in DMEM or EBSS, followed by live ratiometric imaging. LysoTracker Green (50 nM) was added to the medium just before imaging. *Arrows* indicate high 543/458 ratio Keima puncta colocalizing with LysoTracker. Scale bar, 10 μm.

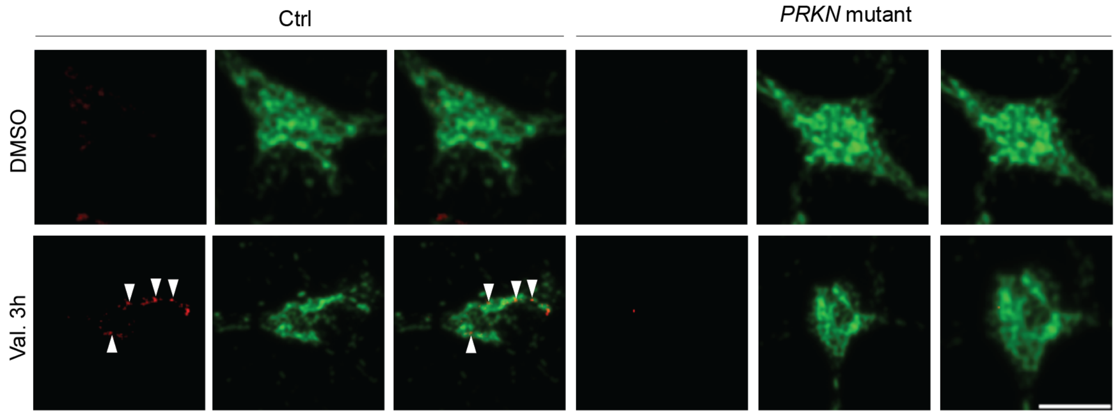

SUPPL. FIG. 4: Detection of endogenous parkin immunoreactivity after mitochondrial depolarization in human control neurons, but not in neurons with *PRKN* mutations. iPSC-derived human control neurons and neurons from a Parkinson's disease patient with compound heterozygous *PRKN* mutations were treated with DMSO or valinomycin (Val., 1 μM) for 3 h and immunostained for endogenous parkin and the mitochondrial marker ATP5F1B, followed by confocal imaging (z stack projections are shown). *Arrowheads* indicate parkin puncta on mitochondria. Scale bar, 10 μm.

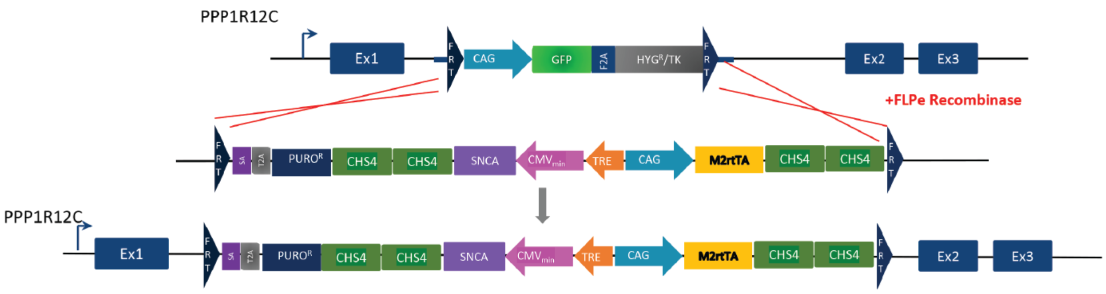

SUPPL. FIG. 5. Schematic of the procedure and constructs used to generate iPSC line carrying a doxycycline-inducible extra *SNCA* copy. A control human iPSC line (iPSC0028) engineered with a recombinase mediated exchange cassette was recombined using FLPe Recombinase with the doxycycline-inducible SNCA containing donor plasmid flanked at both sides by 2 cHS4 insulators. FRT: Flippase recognition target; cHS4: chicken hypersensitive site 4 insulator; SA: splice acceptor; Puro^R: Puromycin resistance gene; HYG^R/TK: hygromycine resistance gene-thymidine kinase gene; CMV$_{min}$: CMV minimal promoter; CAG: CAG promoter; TRE: tetracycline response element; M2rtTA: M2 reverse tetracycline transactivator; PPP1R12C: Protein Phosphatase 1 Regulatory Subunit 12C (gene that contains the *AAVS1* locus in its first intron).

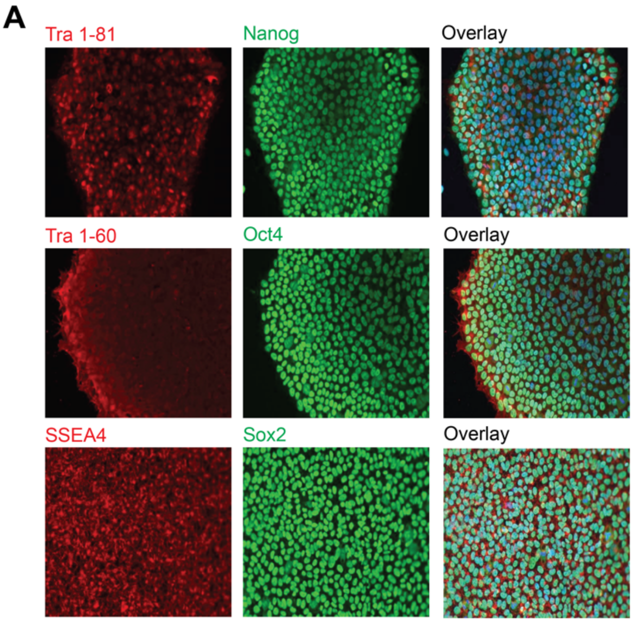

B

	Scorecard Δ Score
Self-renewal	-3.06
Ectoderm	1.97
Mesoderm	1.15
Endoderm	1.31

C

	iPSC0028	iPSC0028 + extra *SNCA* copy
	allele1/allele2	allele1/allele2
C___1563023_10	VIC/FAM	VIC/FAM
C___1801627_20	VIC/FAM	VIC/FAM
C___2728408_10	VIC/FAM	VIC/FAM
C___1250735_20	FAM/FAM	FAM/FAM
C__15935210_10	VIC/VIC	VIC/VIC
C___7431888_10	FAM/FAM	FAM/FAM
C___3227711_10	VIC/VIC	VIC/VIC
C___1902433_10	VIC/FAM	VIC/FAM
C__30044763_10	VIC/VIC	VIC/VIC
C__31386842_10	VIC/FAM	VIC/FAM
C__33211212_10	VIC/FAM	VIC/FAM
C__26524789_10	VIC/FAM	VIC/FAM
C__11821218_10	VIC/FAM	NOAMP
C_____43852_10	VIC/FAM	VIC/FAM
C___1670459_10	VIC/VIC	VIC/VIC
C___8924366_10	FAM/FAM	FAM/FAM
C___1007630_10	FAM/FAM	FAM/FAM
C__11522992_10	FAM/FAM	FAM/FAM
C___7421900_10	VIC/FAM	VIC/FAM
C__10076371_10	VIC/FAM	VIC/FAM
C__26546714_10	VIC/FAM	NOAMP
C___1122315_10	VIC/FAM	VIC/FAM
C__27402849_10	VIC/VIC	VIC/VIC
C___7457509_10	VIC/VIC	VIC/VIC
C__29619553_10	NOAMP	NOAMP
C__11710129_10	VIC/FAM	VIC/FAM
C___2953330_10	VIC/VIC	VIC/VIC
C___1027548_20	UND	UND
C___8850710_10	VIC/VIC	VIC/VIC
C___1083232_10	NOAMP	NOAMP
C__16205730_10	VIC/FAM	NOAMP
C___8938211_20	NOAMP	UND

SUPPL. FIG. 6. Quality control data for iPSC line carrying a doxycycline-inducible extra *SNCA* copy. (A) Immunofluorescence of colonies shows expression of the pluripotency markers TRA-1-81, NANOG, TRA-1-60, OCT4, SSEA4 and SOX2. Nuclei were stained with Hoechst (blue). **(B)** ScoreCard results of trilineage differentiation potential confirmed the capacity to differentiate towards all three germ layers. **(C)** Analysis of genomic identity of the original control iPSC line (iPSC0028) and the iPSC0028 line in which the doxycycline-inducible extra *SNCA* copy had been introduced (iPSC0028 + extra *SNCA* copy) using single nucleotide polymorphism (SNP) analysis and TaqMan Genotyper software.

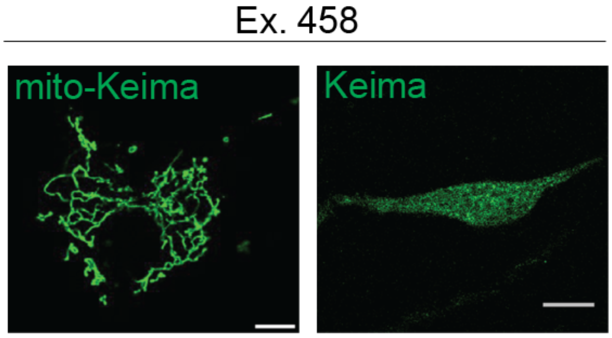

SUPPL. FIG. 7: Mito-Keima and Keima emission images at 458 nm excitation in neurons. Control iPSC-derived neurons on day 50 after neuronal induction were transduced to express mito-Keima or Keima, as indicated, followed by live imaging in basal conditions. Confocal images of emission at 458 nm excitation are shown. Total area of mito-Keima emission at 458 nm excitation was used to determine total mitochondrial area as denominator for the mitophagy index, and total area of Keima emission at 458 nm excitation was used to determine total cell area as denominator for the non-mitochondrial autophagy index. Scale bars, 10 μM.

Uncropped western blots of Fig. 1a

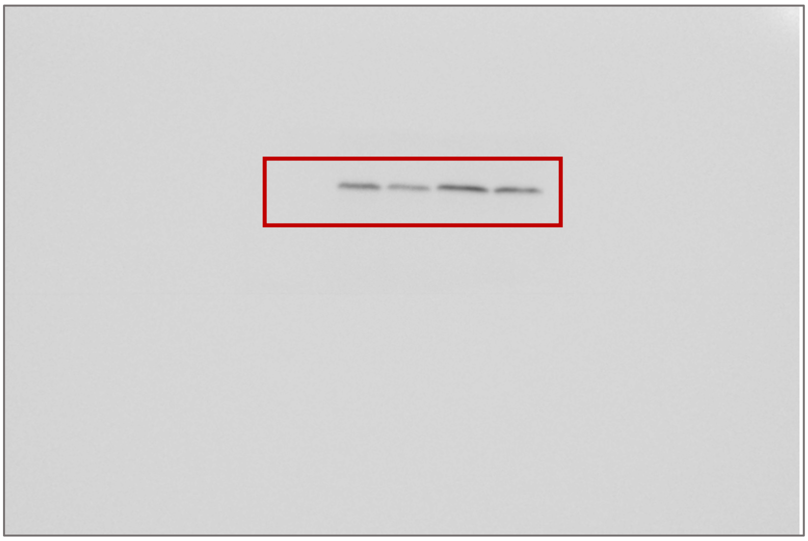

α-synuclein

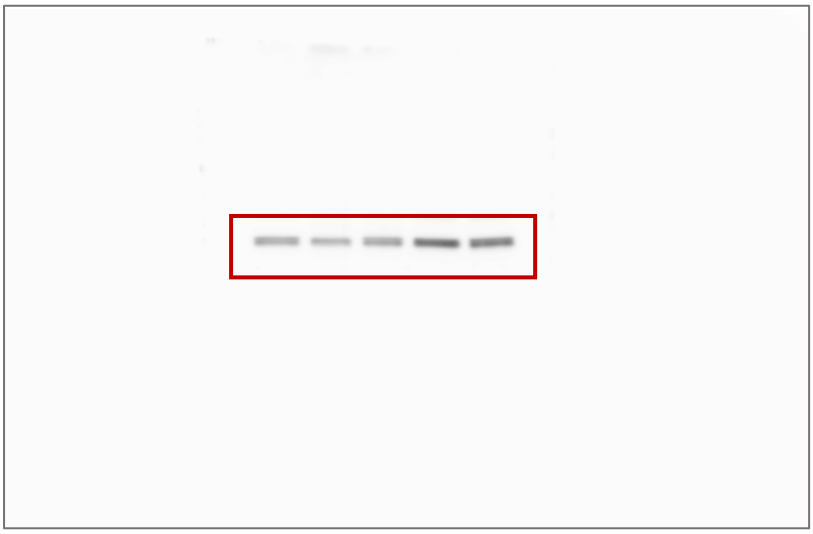

GAPDH

Red boxes indicate cropped areas shown in the figure.

Uncropped western blots of Fig. 3a

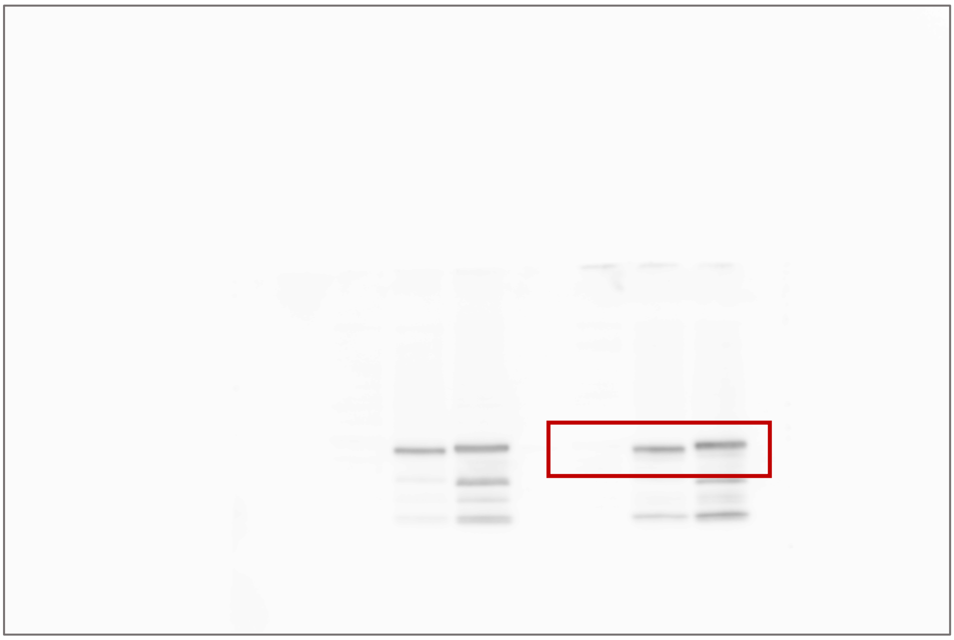

tGFP

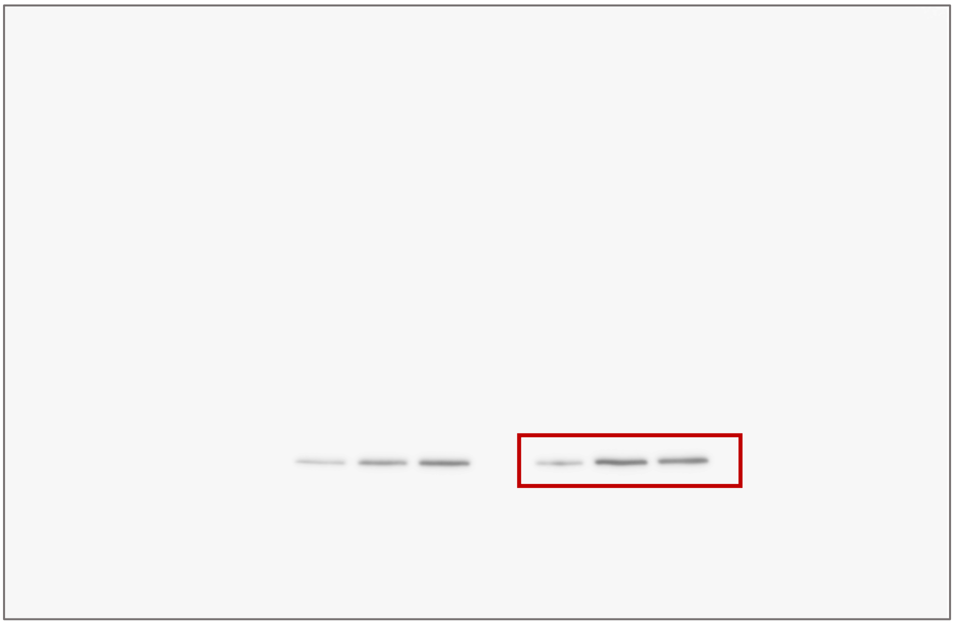

β-actin

Red boxes indicate cropped areas shown in the figure.

Uncropped western blots of Fig. 3d

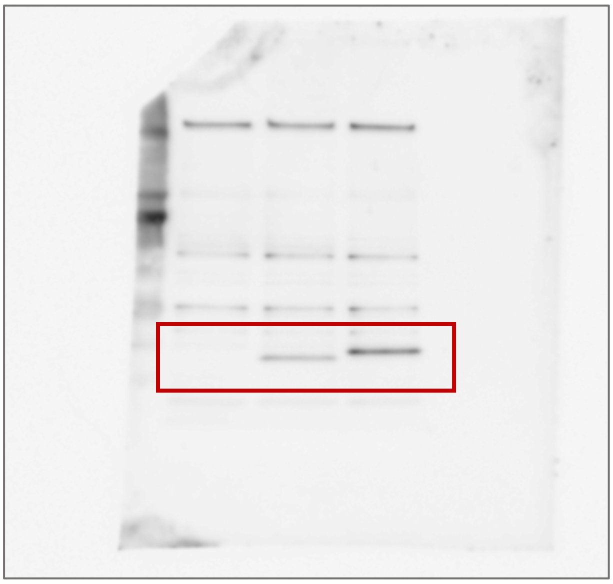

FLAG

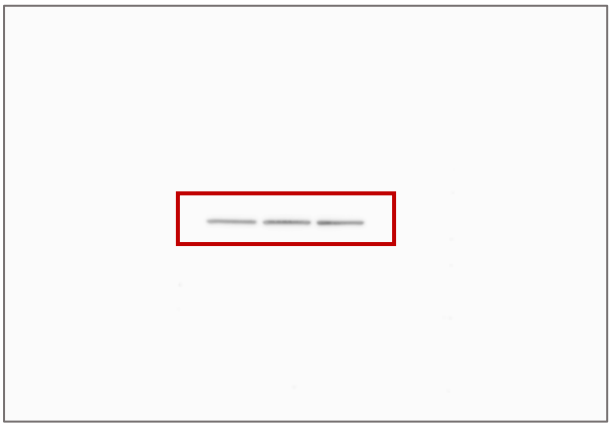

β-actin

Red boxes indicate cropped areas shown in the figure.

Uncropped western blots of Fig. 4d

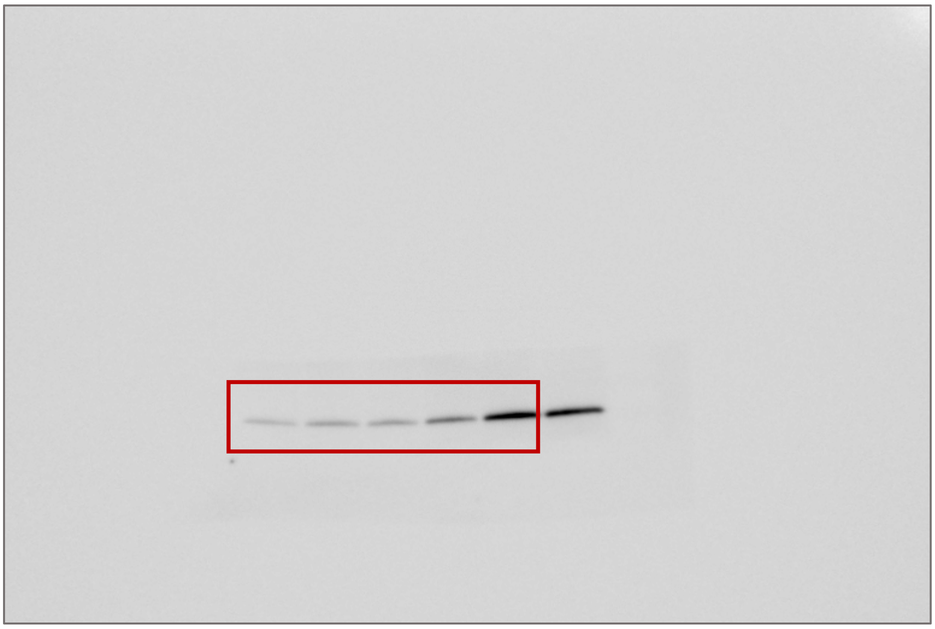

α-synuclein

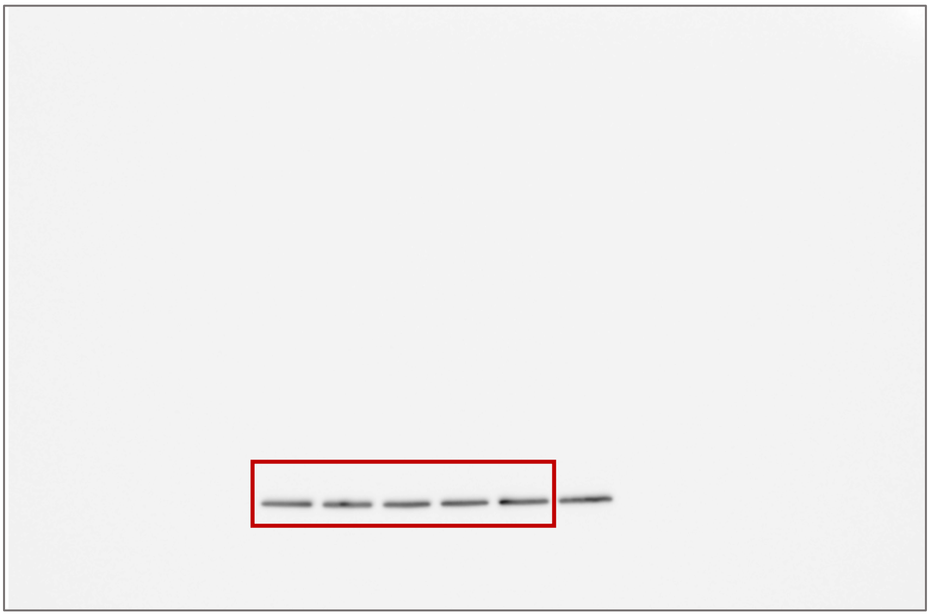

β-actin

Red boxes indicate cropped areas shown in the figure.

Uncropped western blots of Fig. 4f

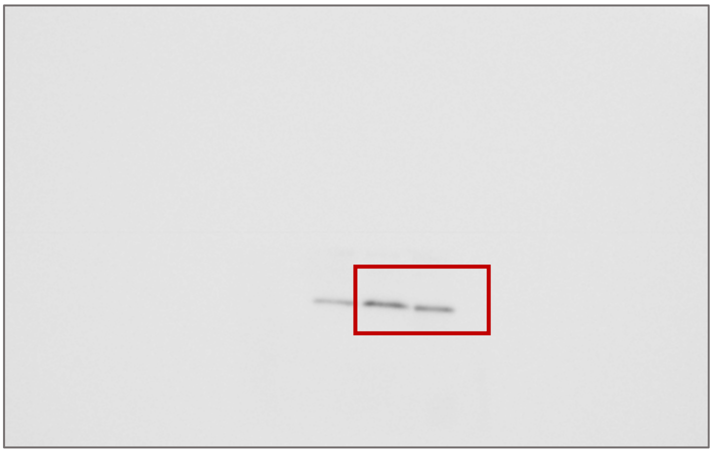

α-synuclein

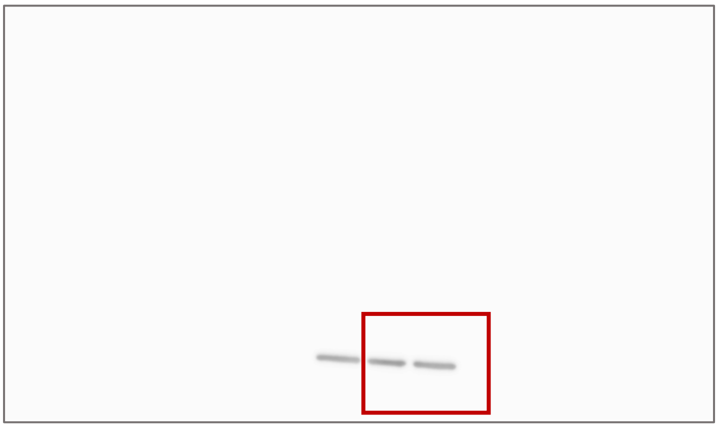

β-actin

Red boxes indicate cropped areas shown in the figure.

Uncropped western blots of Fig. 5a

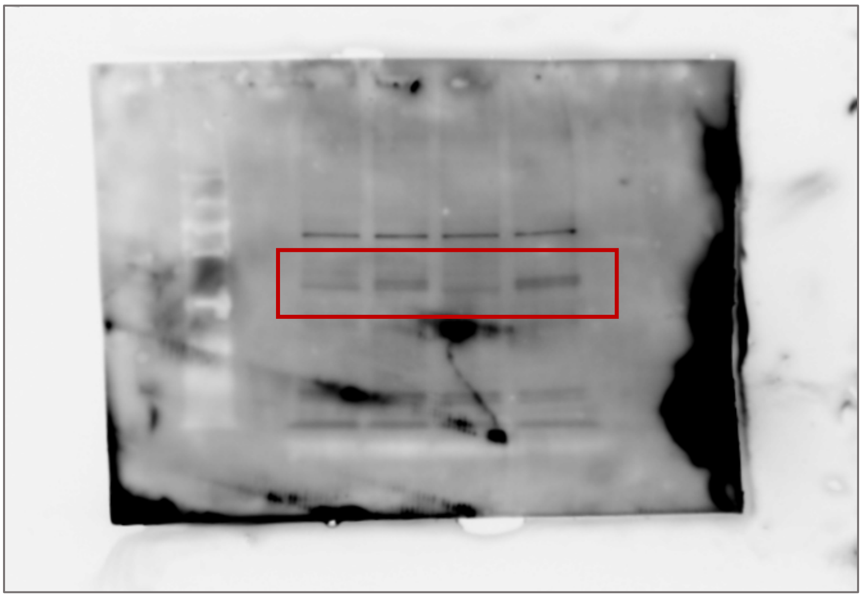

PINK1

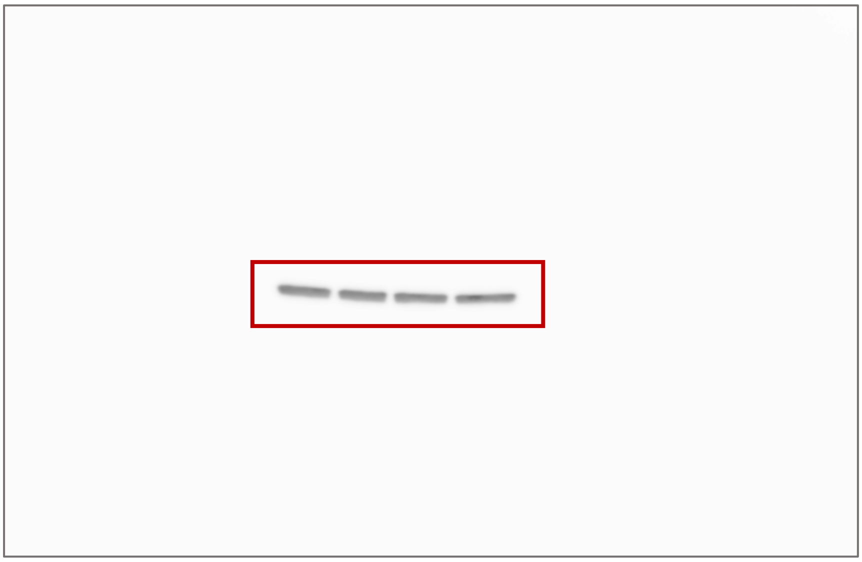

β-actin

Red boxes indicate cropped areas shown in the figure.

Uncropped western blots of Fig. 6c

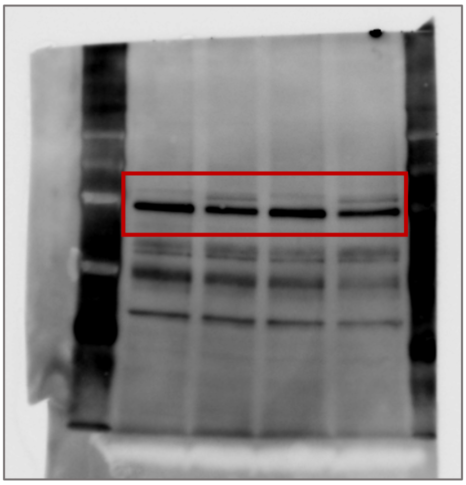

MFN2
Dark exposure

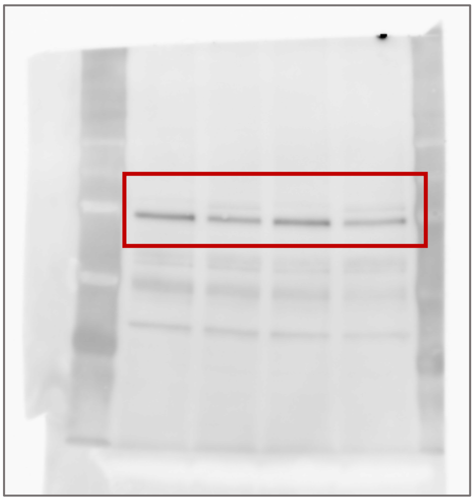

MFN2
Light exposure

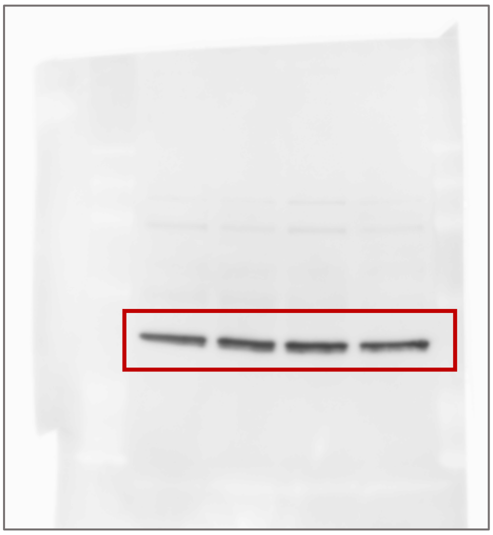

β-actin

Red boxes indicate cropped areas shown in the figure.

Uncropped western blots of Fig. 6f

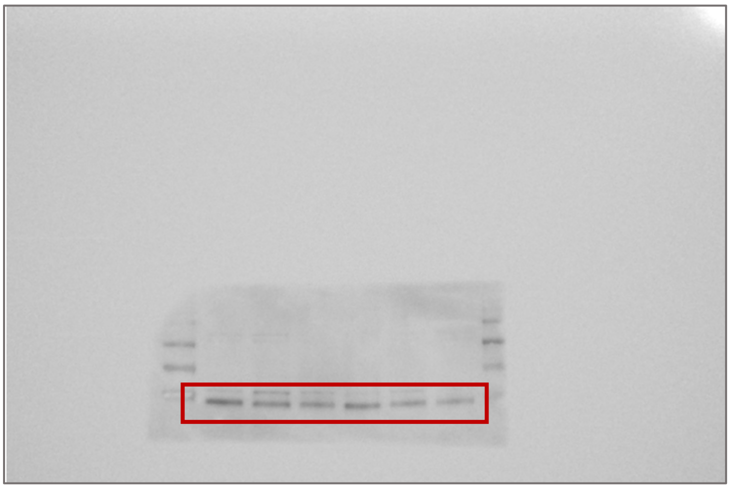

MIRO1

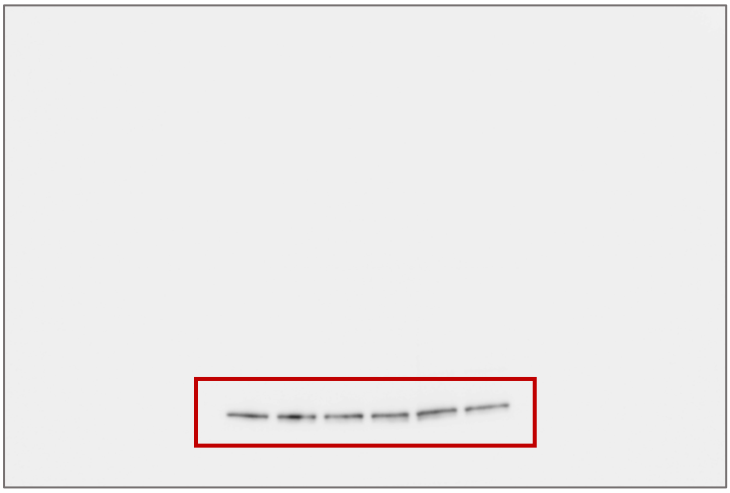

HSP60

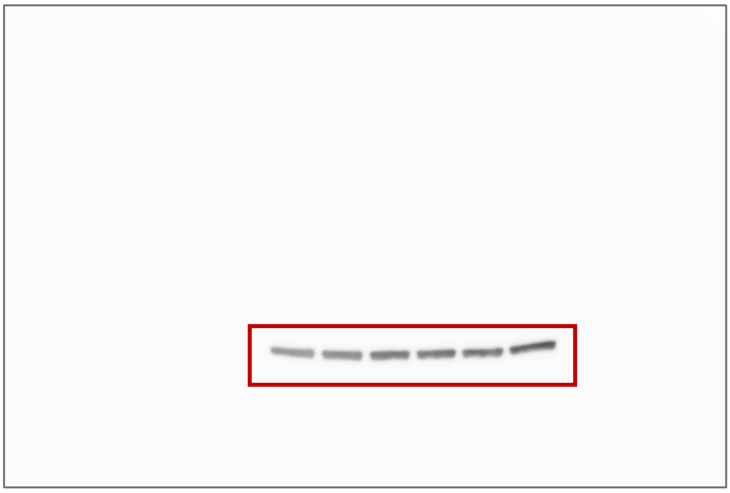

β-actin

Red boxes indicate cropped areas shown in the figure.

Uncropped western blots of Fig. 7a

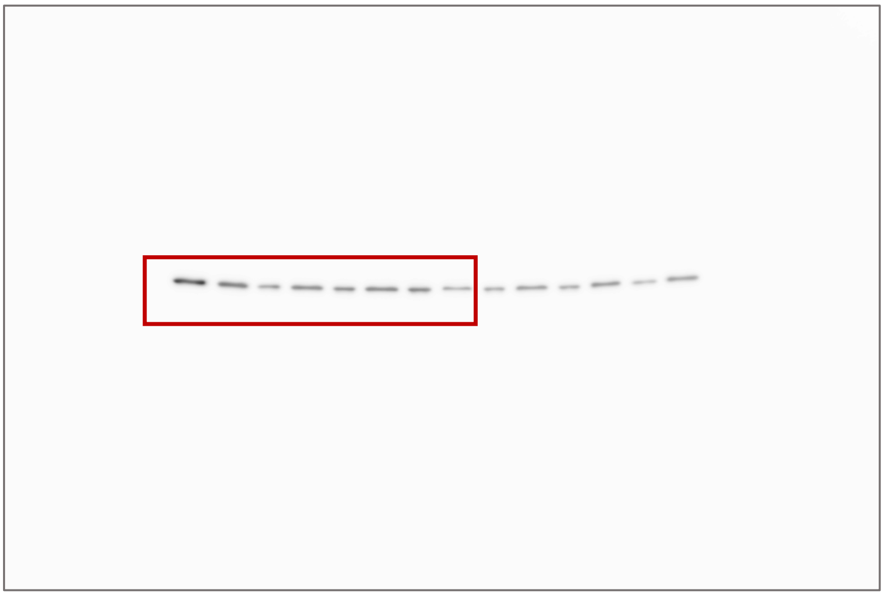

Actin

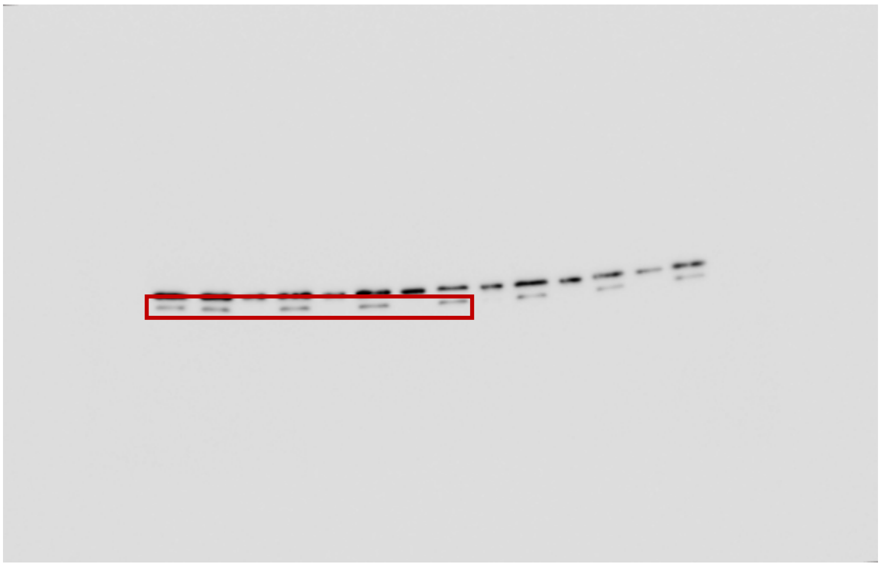

GAPDH
(blot shown above reprobed with anti-GAPDH; upper bands are actin bands that are still visible)

Red boxes indicate cropped areas shown in the figure.

Uncropped western blots of Fig. 7c

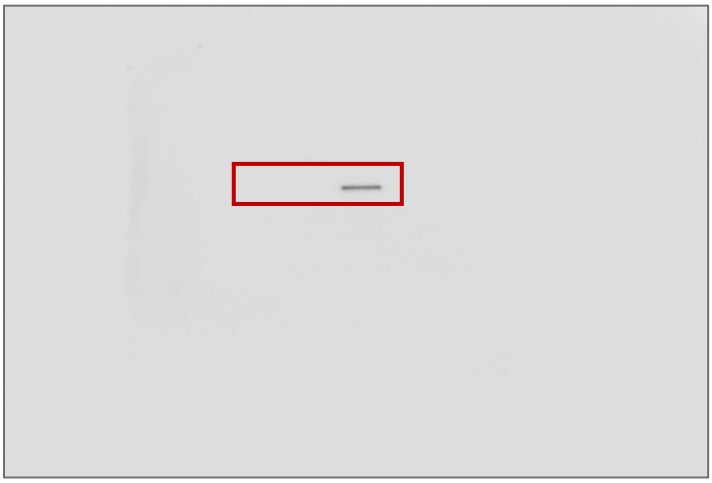

mGFP

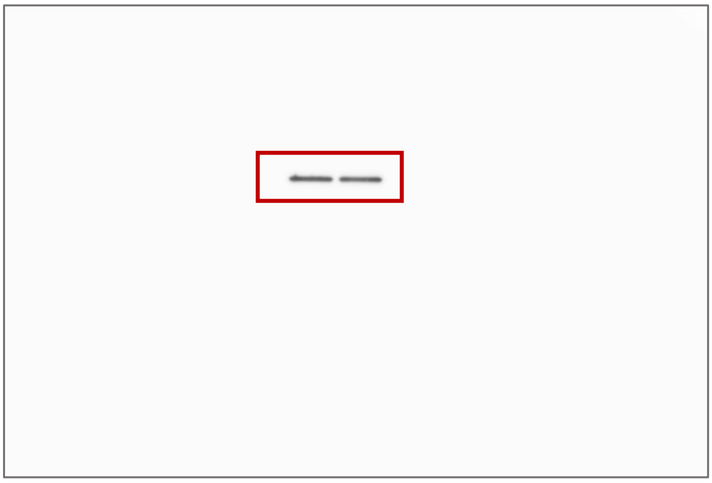

β-actin

Red boxes indicate cropped areas shown in the figure.

Uncropped western blots of Fig. 7f

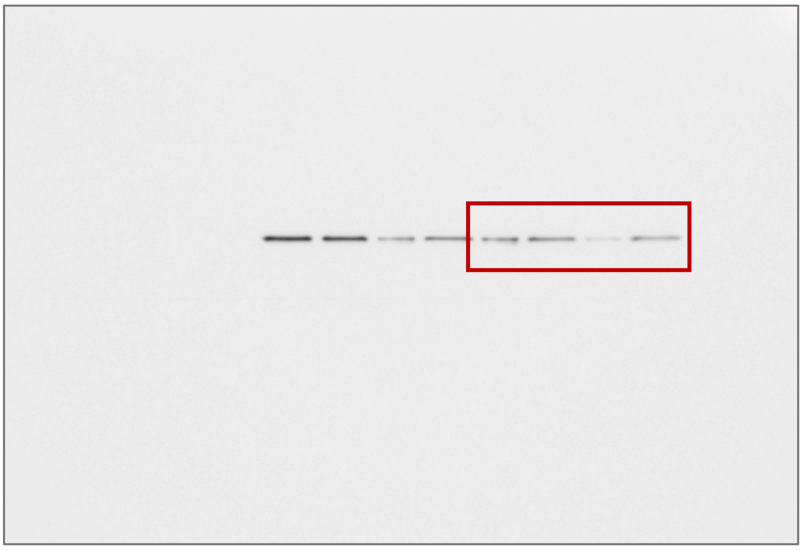

Actin

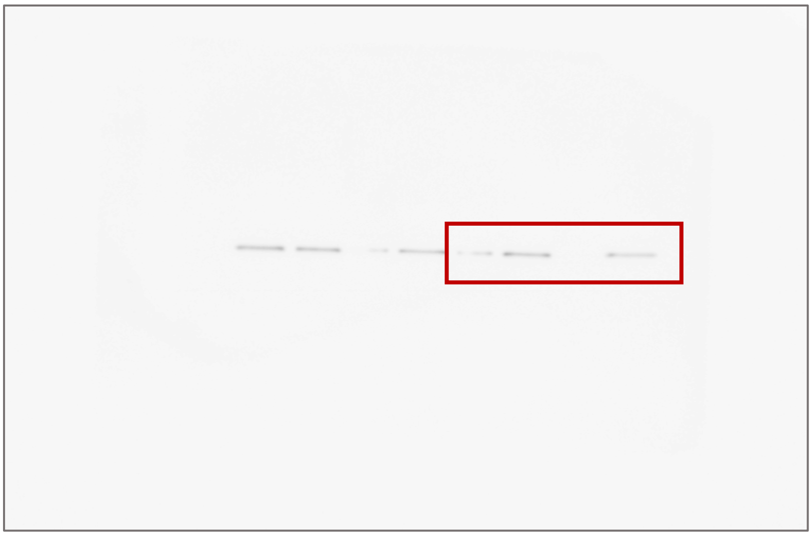

GAPDH

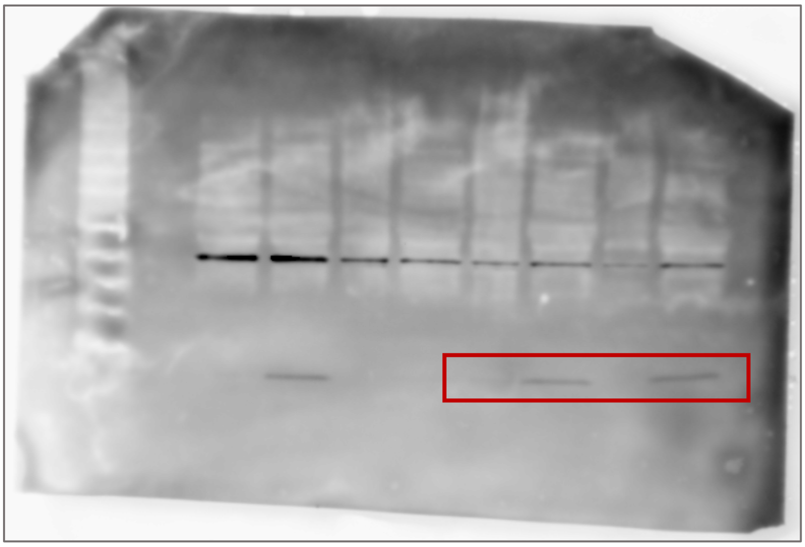

α-synuclein

Red boxes indicate cropped areas shown in the figure.
